# RNA Stabilizes Transcription-Dependent Chromatin Loops Induced By Nuclear Hormones

**DOI:** 10.1038/s41598-019-40123-6

**Published:** 2019-03-08

**Authors:** Antonio Pezone, Candida Zuchegna, Alfonso Tramontano, Antonella Romano, Giusi Russo, Mariarosaria de Rosa, Maria Vinciguerra, Antonio Porcellini, Max E. Gottesman, Enrico Vittorio Avvedimento

**Affiliations:** 1 Dipartimento di Medicina Molecolare e Biotecnologie Mediche, Università di Napoli Federico II, Naples, Italy; 2 Dipartimento di Biologia, Università di Napoli Federico II, Naples, Italy; 3 DNA Metabolism Laboratory, IFOM, The FIRC Institute of Molecular Oncology, Milan, Italy; 4 Institute of Cancer Research, Columbia University Medical Center, New York, NY 10032 USA

## Abstract

We show that transcription induced by nuclear receptors for estrogen (E_2_) or retinoic acid (RA) is associated with formation of chromatin loops that juxtapose the 5’ end (containing the promoter) with the enhancer and the 3′ polyA addition site of the target gene. We find three loop configurations which change as a function of time after induction: 1. RA or E_2_-induced loops which connect the 5′ end, the enhancer and the 3′ end of the gene, and are stabilized by RNA early after induction; 2. E_2_-independent loops whose stability does not require RNA; 3. Loops detected only by treatment of chromatin with RNAse H1 prior to hormonal induction. RNAse H1 digests RNA that occludes the relevant restriction sites, thus preventing detection of these loops. R-loops at the 5′ and 3′ ends of the RA or E_2_-target genes were demonstrated by immunoprecipitation with anti-DNA-RNA hybrid antibodies as well as by sensitivity to RNAse H1. The cohesin RAD21 subunit is preferentially recruited to the target sites upon RA or E_2_ induction of transcription. RAD21 binding to chromatin is eliminated by RNAse H1. We identified E_2_-induced and RNase H1-sensitive antisense RNAs located at the 5′ and 3′ ends of the E_2_-induced transcription unit which stabilize the loops and RAD21 binding to chromatin. This is the first report of chromatin loops that form after gene induction that are maintained by RNA:DNA hybrids.

## Introduction

Four main types of transcription-associated chromatin loops have been described: 1. Intragenic loops, which join promoters and terminators; 2. Enhancer-promoter loops maintained by specific transcription factors and, in some instances, by non-coding RNAs associated with transcription activation; 3. Repressive loops, which downregulate transcription by joining repressor target regions, such as polycomb, with promoters; 4. Insulator loops, which join the ends of individual loci to separate and protect them from the influence of the surrounding genome^1–3^.

Nevertheless, despite the wealth of data on larger scale chromatin domains, such as the Topological Association Domains^4^, we still do not know the precise structure of intragenic loops or the dynamics of their formation after transcription induction.

To approach these problems, we analyzed loop formation and persistence by chromosome conformation capture (3C) after induction of transcription by estrogens (E_2_) or retinoic acid (RA) in synchronized cells. We also investigated whether RNA is involved in loop formation at RA or E_2_-induced genes by digesting chromatin with RNAse H1 and by immunoprecipitation of DNA/RNA hybrids with specific antibodies (DRIP).

We monitored three genes after induction of transcription by nuclear hormones: Caspase 9 (CASP9), a 35 Kb gene induced by RA^5^ and the B-cell lymphoma 2 (BCL2) and Caveolin 1 (CAV1) genes under E_2_ control^6,7^. We first identified the cross-interacting regions of the genes and then focused on the specific intragenic contact regions induced by RA or E_2_.

We excluded other interactions not directly dependent on RA or E_2_ and focused principally on loops that form during RA or E_2_-induced transcription. To study these loops, we selected restriction enzymes that define the most informative interactions of the various genes induced by RA and E_2_ and used these enzymes to detect changes of loop configurations as a function of time after induction.

## Results

### Retinoic acid induces an RNA-stabilized transcription loop in CASP9

To find the relevant chromatin domains assembled in response to RA, we systematically analyzed the structure of CASP9 chromatin by 3C. Chromatin was digested with Nco I, diluted 1:5,000, and ligated. The ligated fragments were detected by real-time PCR or by gel analysis. In most cases, we sequenced the ligated fragments to confirm their location and identity. In parallel samples, chromatin was digested with RNAse H1 prior to ligation.

**Figure 1:**
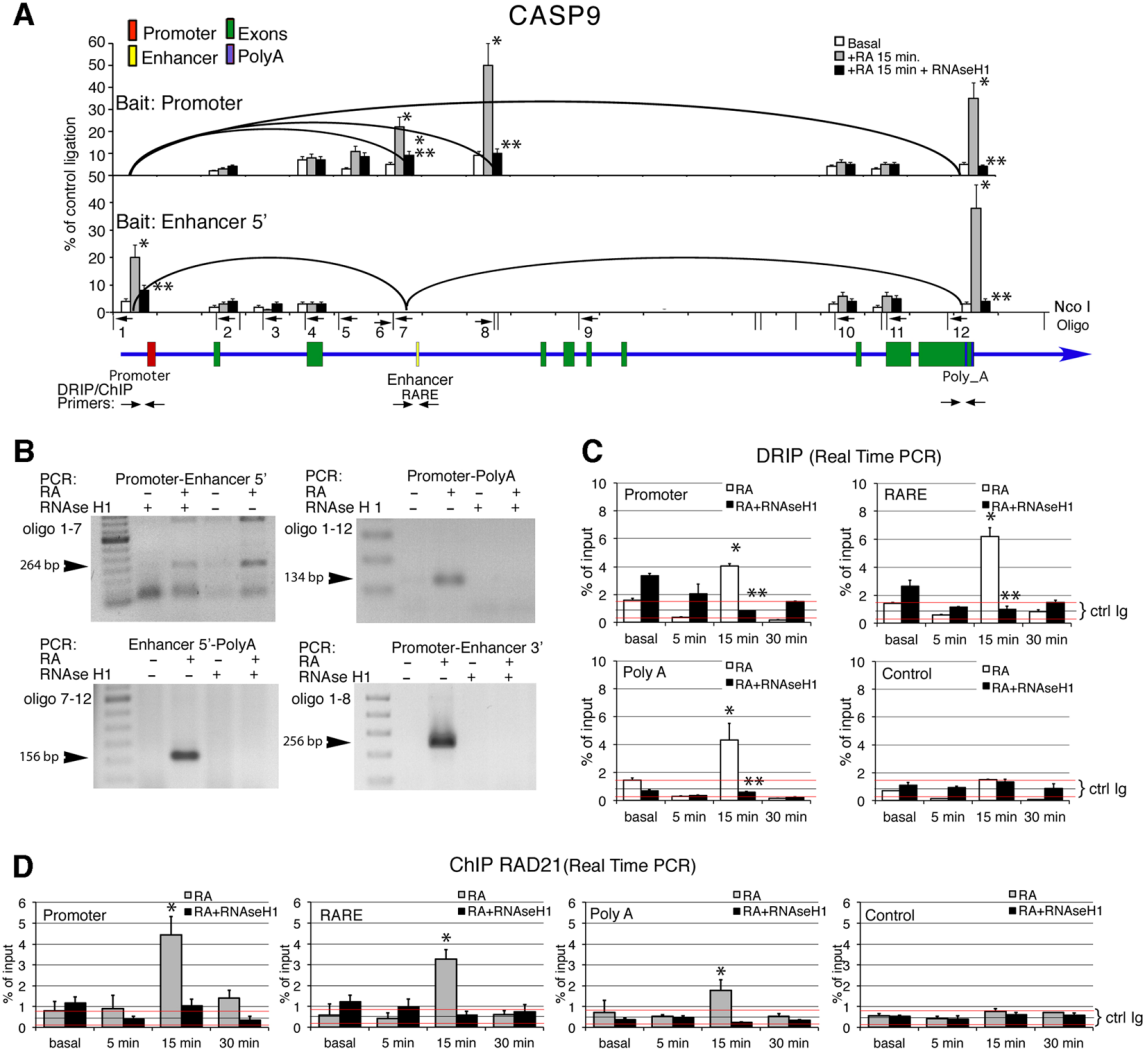
5′-3′ intragenic chromatin loops in CASP9 gene induced by retinoic acid (RA). (**A**) CASP9 gene structure and regulatory elements are shown as colored boxes in a blue line with the arrow indicating the direction of transcription; promoter (red), enhancer (yellow), exons (green), polyA addition sites (black). The black vertical lines indicate the restriction sites used to digest formaldehyde-fixed chromatin from MCF7 breast cancer cells induced for 15 min with 10 nM RA. Numbered horizontal arrows show the primers used to detect specific ligated fragments. The black curved lines show the interactions found in CASP9 chromatin derived from cells exposed to RA, using the promoter (upper panel) or the 5′of the RARE (lower panel) as baits. The bar graphs show the quantification of the interactions measured by 3C analysis (qPCR) between the baits and the primers (arrows and numbers in the lower panel) in the chromatin from unstimulated (basal), RA-stimulated cells for 15 min (RA 15 min) and treated with RNAse H1 for 1 h. Wilcoxon sign-rank test for matched pairs * or **p ≤ 0.001 compared to the basal or to the sample not treated with RNAse H1, respectively. (**B**) 3C analysis of CASP9 intragenic loops. CASP9 ligated fragments were separated by agarose gel electrophoresis (left panel) or identified by qPCR (right panel). The ligated fragments were sequenced to confirm the results obtained by qPCR and electrophoresis. Enhancer regions were detectable both with primers located at 5′ and 3′ end of the Nco I fragment containing the RARE. Where indicated, digestion with RNase H1 (100 U/ml) of formaldehyde-fixed chromatin for 60 min was carried out prior to DNA ligation. The 3C protocol used is described in Materials and Methods and in refs^5,8^. (**C**) DNA-RNA immunoprecipitation (DRIP) analysis of promoter-RARE and polyA sites in CASP9 gene upon RA induction. Cells were induced with RA for different periods of time, DNA was extracted and DRIP analysis was carried as described in Materials and Methods. DNA was treated in parallel samples with RNAse H1 for 1 h before DRIP. Exon 9 of the TSH receptor gene was used as control. The data set corresponding to (**C**) are shown in Supplemental Fig. S1A, *t* test: *p ≤ 0.001 as compared to the basal, Wilcoxon sign-rank test for matched pairs: **p ≤ 0.001 as compared to the sample not treated with RNAse H1. (**D**) Chromatin immunoprecipitation of the RAD21cohesin subunit, at the promoter-RARE and polyA sites in CASP9 gene upon RA induction. Cells were induced with RA for different periods of time, fixed, and the nuclei prepared and subjected to ChIP analysis with antibodies to RAD21. In parallel samples, fixed chromatin was treated with RNAse H1 before ChIP. Exon 9 of the TSH receptor gene was used as control. The data set corresponding to (**D**) are shown in Supplemental Fig. S1B, *t* test: *p ≤ 0.001 as compared to the basal.

CASP9 was scanned with >12 primers using as bait sequences the promoter, the Retinoic Responsive Element (RARE) and the polyA site^5^. The RA-dependent interactions of CASP9 are shown as curved lines connecting the various gene segments with the promoter or RARE (Fig. 1A, upper and lower panel, respectively).

Figure 1A Shows the interactions of the gene domains found in chromatin of cells exposed for 15 min to RA. No other interactions were detected using the primers shown in the figure. Figure 1B shows that upon 15 min RA induction, CASP9 formed several chromatin loops connecting the promoter (red box), the enhancer (RARE, yellow box) and the polyA addition site(s). The loops shown in Fig. 1 represent a unique chromatin domain induced by RA that juxtaposes the promoter-enhancer and CASP9 polyA site.

To further define the configuration of the promoter-polyA loop, we treated formaldehyde-fixed chromatin with RNAse H1 and asked if the loop was sensitive to RNAse H1 digestion. Nuclease sensitivity indicates the loop is stabilized by an RNA- DNA hybrid. Figure 1B shows that RNAse H1 destroyed the RA-induced loop that connects the promoter, RARE, and polyA sites.

To confirm that DNA-RNA hybrids are involved in RA-induced loops, we immunoprecipitated chromatin from RA-induced cells with anti DNA-RNA antibodies (S9.6) and amplified the CASP9 promoter-RARE and polyA sites. Figure 1C and Supplemental Fig. S1A show that 15 min after exposure to RA, we detect R-loops at the promoter, RARE and polyA sites. These loops are eliminated by digestion of chromatin with RNAse H1. With the same timing as R-loop formation, and at the same sites, the RAD21cohesin subunit, accumulated, with a peak at 15 min after RA-induction. The binding of RAD21 to these chromatin sites was inhibited by RNAseH1 (Fig. 1D, Supplemental Fig. S1B).

### Estrogens induce RNA: DNA-stabilized transcription loops in BCL2 and CAV1

**Figure 2:**
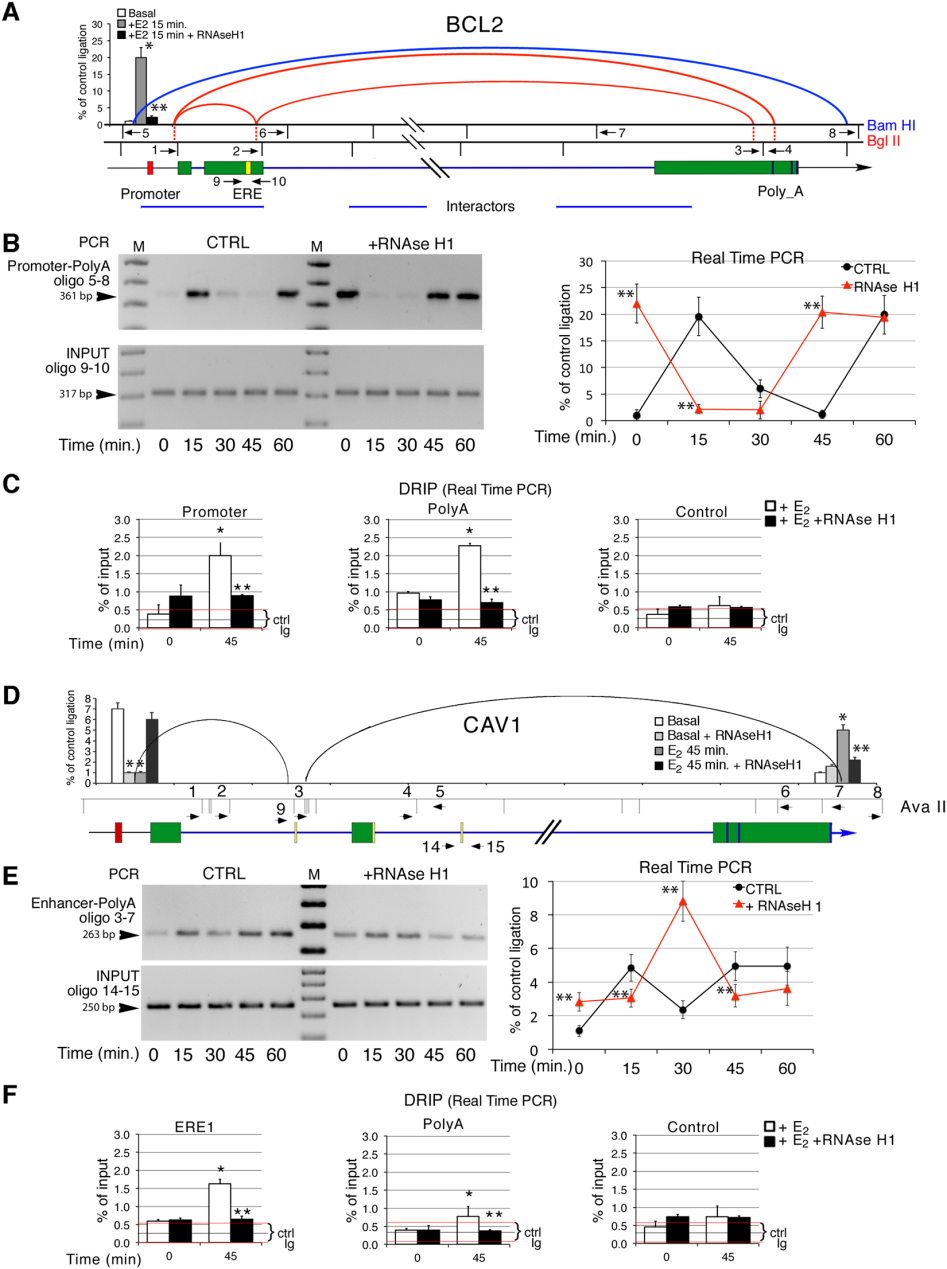
5′-3′ intragenic chromatin loops in BCL2 and CAV1 gene induced by estrogen. (**A**) BCL2 gene structure and regulatory elements. The black vertical lines indicate the restriction sites used to digest formaldehyde-fixed chromatin after induction with 10 nM E_2_ for various periods of time (min). Horizontal arrows indicate the primers used to detect specific ligated fragments. All primer combinations were tested using the promoter (oligonucleotide 1 or 5), the ERE (oligonucleotides 6 or 2) and polyA site (oligonucleotide 8 and 4) as bait sequences. The red and the blue curved lines show interactions in BCL2 chromatin derived from cells exposed to E_2_ digested with Bam HI (blue) or Bgl II (red). The blue lines show interacting regions identified by paired-end tag sequencing (ChIA-PET), which detects global chromatin interactions of estrogen receptor α (ERα)^8^. E_2_-dependent interactions were selected for further analysis with RNAse H1. The histograms show the quantification by qPCR of the promoter-polyA loop (oligonucleotides 5 and 8) under various conditions. Non-parametric Wilcoxon sign-rank test for matched pairs: * or **p ≤ 0.001 compared to the basal or to the sample not treated with RNAse H1, respectively. (**B**) BCL2 ligated fragments separated by agarose gel electrophoresis (left panel) or identified by qPCR (right panel). The primers used to detect the ligated fragment are indicated on the left side of the gel. INPUT shows the input DNA in the 3C experiment; a PCR fragment amplified with primers 9 and 10 containing the ERE. Sequencing the ligated fragments confirmed the qPCR and electrophoresis results. Where indicated, digestion with RNase H1 (100 U/ml) of formaldehyde-fixed chromatin for 60 min was carried out before DNA ligation. The right panel shows the quantification by qPCR of the promoter-polyA loop shown in the left hand side gel. Non-parametric Wilcoxon sign-rank test for matched pairs: * or **p ≤ 0.001 compared to the basal or to the sample not treated with RNAse H1, respectively. (**C**) DNA-RNA immunoprecipitation (DRIP) analysis of promoter and polyA sites in BCL2 gene upon E_2_ induction. Cells were induced with E_2_ for 45 min, DNA was extracted and DRIP analysis was carried as described in Materials and Methods. DNA was also treated with RNAse H1 for 1 h before immunoprecipitation. Exon 9 of the TSH receptor gene was used as control. The data set corresponding to Fig. 2C are shown in Supplemental Fig. S2A, *t* test: *p ≤ 0.001 ascompared to the basal, Wilcoxon sign-rank test for matched pairs: **p ≤ 0.01 as compared to the sample not treated with RNAse H1. (**D**) CAV1 gene structure and regulatory elements. The black vertical lines indicate the Ava II restriction sites used to digest formaldehyde-fixed chromatin of cells exposed to 10 nM E_2_ for various periods of time (min). Horizontal arrows indicate the primers used to detect specific ligated fragments. All combinations of the primers were tested using the promoter (oligonucleotide 1 and 2), the ERE (oligonucleotides 9, 3, 4 and 5) and the polyA site (oligonucleotide 7 and 8) as bait sequences. The black curved lines show the interactions found in CAV1 chromatin derived from cells treated with E_2_ and digested with Ava II. E_2_-dependent interactions were selected for testing with RNAse H1. The bar graphs at the left and right of CAV1 gene show the quantitative analysis of the promoter-ERE (primers 1–9) and ERE-polyA (primers 3–7) loops respectively. The ANOVA test on the time course is shown in Supplemental Fig. S2B. (**E**) CAV1 ligated fragments separated by agarose gel electrophoresis (left panel) or identified by qPCR (right panel). The primers used to detect the ligated fragment are indicated on the left side of the gel. INPUT indicates the PCR of the fragment amplified by primers 14 and 15 containing the ERE. The ligated fragments were sequenced to confirm the qPCR and electrophoresis results. Where indicated, digestion with RNase H1 (100 U/ml) of formaldehyde-fixed chromatin for 60 min was carried out before DNA ligation. The right panel shows the quantification by qPCR of the ERE-polyA loop shown on the gel on the left side. Non-parametric Wilcoxon sign-rank test for matched pairs: * or ** p ≤ 0.001 compared to the basal or to the sample not treated with RNAse H1, respectively. The ANOVA test on the time course is shown in Supplemental Fig. S4A. We found no interaction between the following CAV1 pairs of primers on restricted chromatin derived from E_2_-stimulated cells: 1 + 2; 1 + 4; 1 + 5; 1 + 6; 1 + 7; 1 + 8; 2 + 3; 2 + 4; 2 + 5; 2 + 6; 2 + 7; 2 + 8; 3 + 4; 3 + 5; 3 + 6; 7 + 7; 3 + 8; 4 + 6; 4 + 7; 4 + 8; 5 + 6; 5 + 7; 5 + 8; 6 + 8 (Supplemental Table 1). (**F**) DNA-RNA immunoprecipitation analysis of promoter and polyA sites in CAV1 gene upon E_2_ induction. Cells were induced with E_2_ for 45 min, DNA was extracted and DRIP analysis was carried as described in Materials and Methods. Parallel DNA samples were treated with RNAse H1 for 1 h before immunoprecipitation. Exon 9 of the TSH receptor gene was used as control. The data set corresponding to Fig. 2F are shown in Supplemental Fig. S4B, *t* test: *p ≤ 0.001 ascompared to the basal, Wilcoxon sign-rank test for matched pairs: **p ≤ 0.01 as compared to the sample not treated with RNAse H1.

To extend our observations to another nuclear hormone, we studied E_2_-induced chromatin loops in two genes: BCL2 and caveolin 1 (CAV1). BCL2, which spans ~200 Kb of genomic DNA, revealed chromatin interactions using restriction enzymes Bam HI and Bgl II (Fig. 2A, blue and red curves, respectively). The interacting segments correspond to the promoter, the Estrogen Responsive Element (ERE) and the polyA site. The same regions, shown in Fig. 2A were found associated by paired-end tag sequencing (ChIA-PET) induced by active estrogen receptor α (ERα) (blue lines in Fig. 2A)^8^.

The sensitivity of the BCL2 promoter-polyA loop to RNAse H1 changed as a function of time after induction (Fig. 2B). The loop was eliminated by RNAse H1 at 15 min., but became resistant to the enzyme at 60 min (Fig. 2B), suggesting that R-loops are present in the early phase of E_2_-induced transcription. At 45 min the loop was detected only after treatment of chromatin with RNAse H1 (Fig. 2B), implying that an R-loop prevented loop detection. To test the presence of RNA:DNA hybrid in the promoter-polyA loop, we immunoprecipitated chromatin from E_2_-induced cells with anti DNA:RNA hybrid antibody (S9.6) and amplified the BCL2 promoter and polyA site. Figure 2C and Supplemental Fig. S2A show R-loops at the promoter and polyA sites at 45 min, which are eliminated by digestion of chromatin with RNAse H1. The RAD21-cohesin subunit accumulated at the same sites and with the same kinetics as R-loop formation, with a peak at 45 min after E_2_-induction. RAD21 binding to these chromatin sites was eliminated by RNAse H1 (Supplemental Fig. S3).

The detection of R-loops at the promoter-polyA regions with anti DNA-RNA antibody complements the data of Fig. 2B. 3C revealed the loop at 45 min only after treatment of chromatin with RNAse H1. This suggests that the restriction site used to cleave the DNA is occluded by RNA and cannot be cleaved and ligated (see below). This loop under these conditions cannot be detected by 3C (without RNAse H1 digestion) but only by DNA-RNA hybrid immunoprecipitation (Fig. 2C, Supplemental Fig. S2A). Collectively, these data show that the promoter and polyA R-loops induced by E_2_ are assembled in a unique structure that juxtaposes the 5′ and 3′ end of BCL2 gene.

We also analyzed the E_2_-induced loops in CAV1, which spans 20 Kb of genomic DNA, by scanning the gene with 15 primers using as bait sequences the promoter and ERE regions. Only the primers located near the promoter, the ERE and the polyA site showed E_2_-dependent interactions. We detected 2 loops: a loop connecting the promoter to the fragment containing the ERE and a loop connecting the ERE with the polyA site (Fig. 2D, Supplemental Fig. S2B). The ERE-polyA loop cycles over time during the early phase of E_2_-induced transcription (Fig. 2E, Supplemental Fig. S4A). It peaked at 15 min, decreased at 30 min, and rose after 45–60 min of E_2_-stimulation. Sensitivity of the CAV1 loop to RNAse H1 varied with the time after induction. At 15 and 45 min the loop was eliminated by RNAse H1, but at 60 min the loop was resistant to digestion (Fig. 2E, Supplemental Fig. S4A). At 45 min, the loop was also detected by immunoprecipitation with anti DNA-RNA antibodies and was sensitive to RNAse H1 (Fig. 2F, Supplemental Fig. S4B).

The CASP9, BCL2 and CAV1 genes behave similarly upon hormone induction; all form loops connecting the promoter, enhancer and polyA sites very early after hormonal induction and are all stabilized by RNA. In all three genes, RAD21 is recruited early after induction of transcription and its binding is dependent on RNA (Fig. 1D and Supplemental Fig. S3). Loop formation is independent of gene size or distance between the ERE or RARE elements to the promoter or polyA sites.

### The E2-induced CAV1 loops mark the 5′ and 3′ ends of specific transcripts

The three genes we have studied are induced by many factors and hormones. They encode several transcripts with different 5′ or 3′ ends, some of which are induced by RA or E_2_ (refs^5,7,9^). We thought it possible that transcription loops induced by RA or E_2_ may direct the selection of 5′ or 3′ ends of the transcript(s).

**Figure 3:**
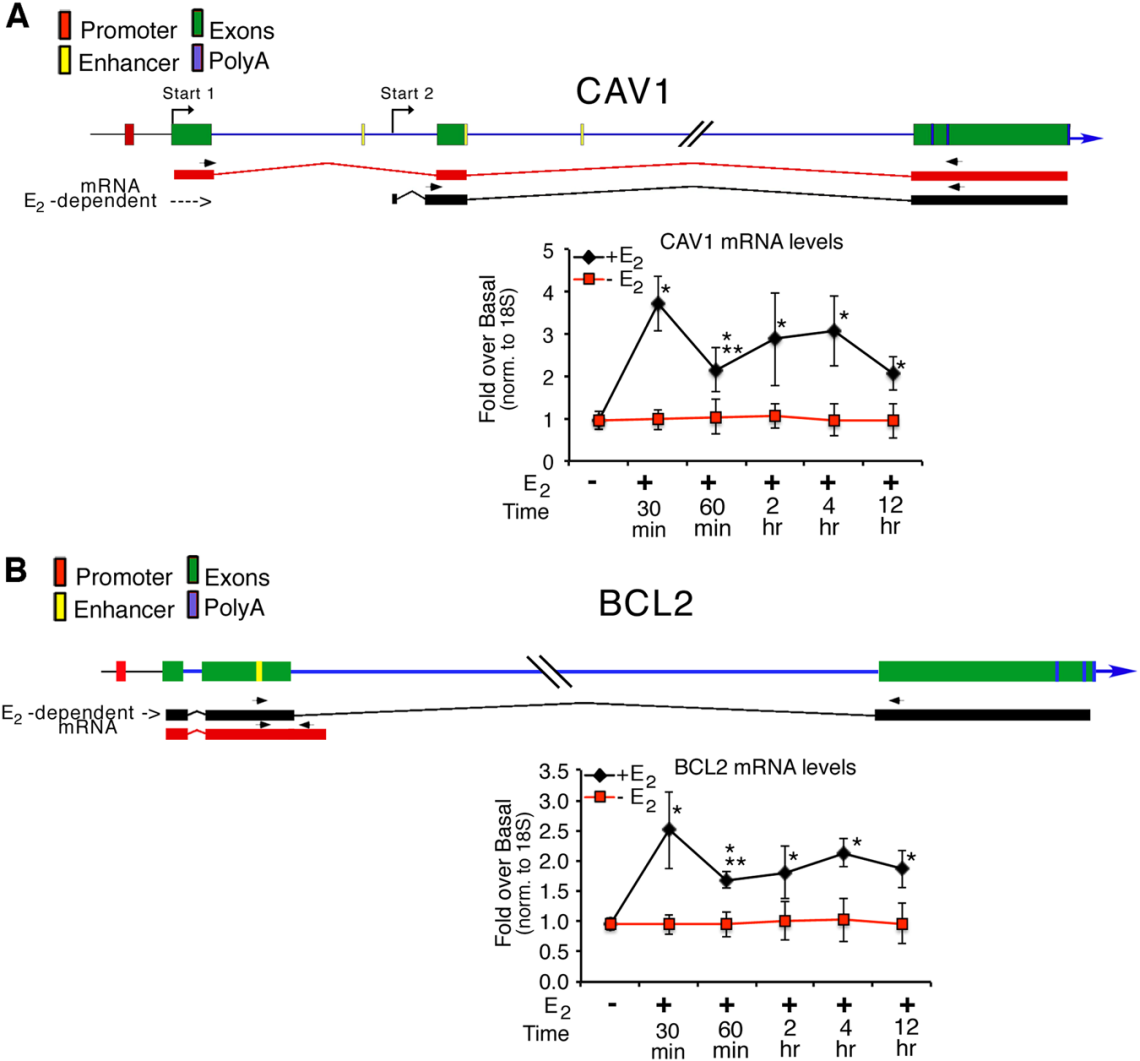
The CAV1 basal and E_2_ induced transcripts differ at their 5′ ends; the BCL2 basal and E_2_-induced transcripts differ at their 3′ ends. (**A**) CAV1 and the CAV1 mRNAs. The top panel shows the genomic structure of the CAV1 gene (red box, promoter for Start 1; green boxes, exons; blue lines, introns; yellow boxes, enhancers (EREs); blue box, polyA sites) and the E_2_-induced transcripts, (black lines, Start 2) and basal transcripts (red lines, Start 1). Primer for start two is shown in Supplementary Table 1 (CAV1-RNAF). This primer detects CAV1 mRNAs indicated as 202 and 203 in ref.^9^. The lower panel shows the basal CAV1 mRNAs in MCF7 cells and the accumulation CAV1 mRNAs after induction, using qPCR, *t* test: * or **p ≤ 0.001 as compared to the basal or 30 min +E_2_, respectively. (**B**) BCL2 and BCL2 mRNAs. The top panel shows the genomic structure of the BCL2 gene (red box, promoter; green boxes, exons; blue lines, introns; yellow box, enhancers (EREs); blue box, polyA sites) and the E_2-_induced (black lines) or basal transcripts (red lines). Primer for the long transcript originating at start 1 is shown in Supplemental Table 1 (BCL2-RNAR).The lower panel shows basal BCL2 mRNAs and accumulation of E_2_-dependent BCL2 mRNAs in MCF7 cells by qPCR, *t* test: * or **p ≤ 0.001 as compared to the basal or 30 min +E_2_, respectively.

**Figure 4:**
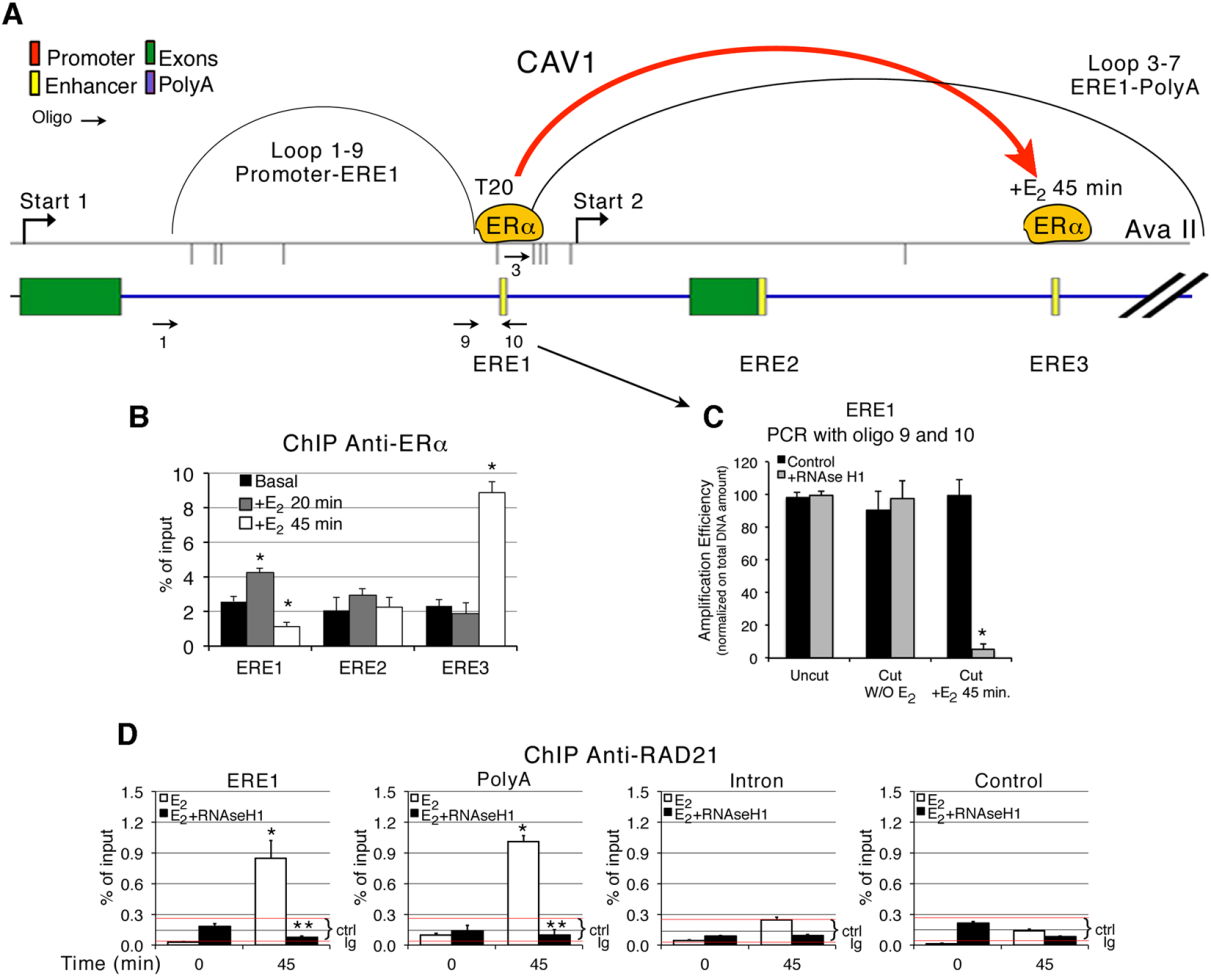
The CAV1 promoter-ERE loop changes configuration after E_2_ -induction. (**A**) Enlarged map of CAV1. Shown are the 3 mapped ERE sites and the E_2_-independent (Start 1) and E_2_-dependent (Start 2) transcription starts (Porcellini *et al*., in preparation and ref.^9^). The curved lines indicate the promoter-ERE loop (identified by oligonucleotides 1 and 9, black arrows) and the ERE-PolyA loop (identified by oligonucleotides 3 and 7, as shown in Fig. 2C). The red curved line represents the localization of the E_2_ receptor (ERα) at 20 and 45 min post E_2_-induction. (**B**) ChIP analysis of ΕRα at the ΕRΕ1, ERE2, ERE2 and ERE3 sites shown in (**A**). The data set corresponding to (**B**) are shown in Supplemental Fig. S5A, *t* test: *p ≤ 0.001 as compared to the basal. (**C**) Restriction of fixed DNA-chromatin with Ava II isolated from cells before or after exposure to E_2_ for 45 min. Parallel samples were treated before DNA isolation with RNAse H1 for 1 h. Digestion was monitored by qPCR with oligonucleotides 9 and 10 (arrow), shown in (**A**), *t* test: *p ≤ 0.001 as compared to the basal. (**D**) Chromatin immunoprecipitation of the RAD21 cohesin subunit at the ERE1, polyA and intron II sites in CAV1 upon 45 min induction with E_2_. Parallel samples were treated with RNase H1 for 30 min. before ChIP. Exon 9 of the TSH receptor gene was used as control. The data set corresponding to (**D**) are shown in Supplemental Fig. S5B, *t* test: *p ≤ 0.001 ascompared to the basal, Wilcoxon sign-rank test for matched pairs: **p ≤ 0.01 as compared to the sample not treated with RNAse H1.

The CAV1 basal and E_2_-induced transcripts have different 5′ ends, whereas the BCL2 basal and E_2_-induced transcripts differ at their 3′ termini (Fig. 3A,B and ref.^9^). To study the relation between the 5′ end of the CAV1 transcripts and the loops shown in Fig. 2D, we analyzed the configuration(s) of the 5′ end loop connecting the CAV1 promoter with the ERE (Fig. 2D). Fine mapping of the E_2_-dependent and independent transcription start sites (TSS) in CAV1 (Fig. 3A) indicated that the E_2_-induced transcript initiates at Start 2, which is located 1.45 Kb 3′ downstream of the E_2_-independent TSS (Start 1, Fig. 4A, ref.^9^). Start 2 is located at the 5′ end of the loop connecting the ERE (ERα in Fig. 4A, yellow box) with the polyA site and excludes the promoter (Fig. 4A) and exon 1. The structure of the CAV1 genomic segment indicates that the ERE-polyA loop in CAV1 has the same configuration as the promoter-polyA loop described in CASP9 and BCL2 (Figs 1A, 2A,D, 4A) connecting the 5′ end (Start 2) and the 3′ end of the transcription unit.

CAV1 includes three ERE sites (Fig. 4A, yellow boxes). Each ERE is able to induce E_2_-dependent CAV1 transcription independently (A. Porcellini, in preparation). ERE1 is upstream of the E_2_-dependent Start 2 and is the preferred site at which the E_2_ receptor, ERα, accumulates during the early phase of induction (Fig. 4A,B and Supplemental Fig. S5A). At 45 min, ERα accumulates at ERE3 (Fig. 4A,B and Supplemental Fig. S5A). Recruitment of the receptor at ERE1 is associated with the inhibition of the promoter-ERE1 loop (detected by oligonucleotides 1–9 in Fig. 2D), which is present prior to induction (Fig. 2D and Supplemental Fig. S2B). Following E_2_-induction, the promoter-ERE1 loop was detectable only after RNAse H1 digestion (Fig. 2D, bar graph, left side of the figure). We conclude that this loop is present in two configurations: 1. associated with basal transcription and destroyed by RNAse H1 and 2. E_2_-dependent and revealed only by RNAse H1 (Fig. 2D and Supplemental Fig. S2B). In the absence of E_2_, the RNA: DNA hybrid stabilizes the loop and favors transcription from Start 1 (Figs 2D and 4A). Following E_2_ induction, RNA hybridized to DNA prevents loop detection or, alternatively, formation. We show that the former is the case; RNA blocks access of the defining restriction enzyme, thus, preventing loop detection. This was confirmed by digesting CAV1 chromatin at the ERE1 site with Ava II in the presence or absence of RNAse H1. We specifically analyzed the site between oligonucleotides 9 and 10, spanning the fragment that marks the 3′ end of the promoter-ERE loop (Figs 2D and 4A). PCR analysis showed that Ava II efficiently cleaves the fragment only after RNAse H1 digestion and only in cells exposed to E_2_, mirroring the conditions that permit the detection of the promoter-ERE loop (Figs 2D and 4C). These data suggest that E_2_-induction is associated with recruitment of ERα to ERE1 and with the appearance of a novel RNA. This RNA hybridizes with DNA at the Ava II site, preventing detection of the promoter-ERE loop, which includes the TSS of the E_2_-independent transcript (Fig. 4A). Under the same conditions (E_2_-induction), the loop linking ERE1 to the polyA site became detectable (Fig. 2C,D), suggesting that the E_2_-bound receptor switches the CAV1 TSS from Start 1 to Start 2 by changing the 5′ end of the ERE1-polyA loop (oligonucleotides 3–7 in Figs 2D and 4A). Induction by E_2_ of the ERE1-polyA loop site was associated with the recruitment of the RAD21 cohesin subunit to the ERE1-polyA sites (Fig. 4D and Supplemental Fig. S5B). Note that RAD21 binding to the ERE1 and polyA sites was eliminated by RNAse H1 (Fig. 4D and Supplemental Fig. S5B).

The BCL2, 5′-polyA and CAV1 ERE-polyA loops cycle over time (Figs 2B,E and Supplemental Fig. S4A). We asked whether these cycles correspond to oscillations in transcriptional activity. We measured the levels of E_2_-induced transcripts and found that E_2_-induced CAV1 and BCL2 mRNA levels peaked at 30 min, fell at 60 min and peaked again at 4 hr, with a delay relative to the timing of the loops of 15 min (Fig. 3A,B). Loop formation and mRNA accumulation mirror the cycling pattern of hormone-induced chromatin re-modeling, as shown by histone H3 methylation and demethylation, and BER and NER enzyme recruitment at the promoters TSS-RARE/ERE-polyA of CASP9^5^, BCL2^7^ and CAV1 (A. Porcellini *et al*., in preparation). These early cycles are critical for transcription initiation, since inhibition of histone demethylation prevents hormone- dependent loop formation and transcription^5,7^. Eventually, cells desynchronize, and oscillations of the indicated parameters were no longer detectable above background

### Estrogen induces antisense, RNAse H1-sensitive, transcripts from the 5′ and 3′ ends of CAV1

The data shown in Figs 2D, 4A,C, indicate that the promoter-ERE loop at the 5′ end of CAV1 is revealed by RNAse H1at certain times following E_2_-induction. The RNAse H1-sensitive configuration might be dependent on a novel E_2_-induced RNA, which hybridizes with the CAV1 segment upstream of Start 2 (Fig. 4A).

**Figure 5:**
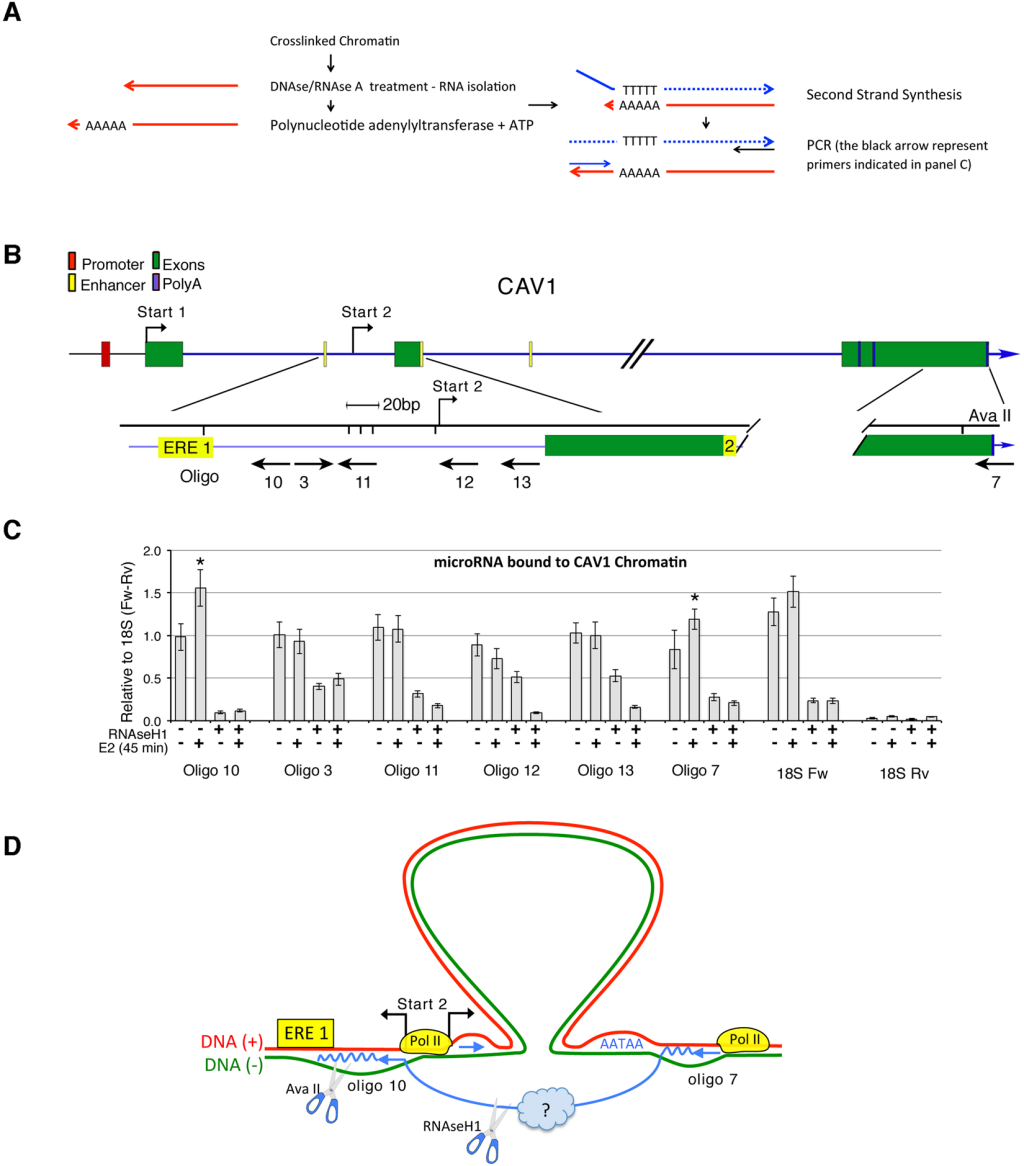
Localization of E_2_-induced sense and antisense chromatin-associated CAV1 RNA. (**A**) Protocol used to isolate and identify sense and antisense RNAs bound to chromatin. An aliquot of formaldehyde–fixed chromatin, isolated for 3C analysis, from E_2_-exposed and control cells was treated with DNAse/RNAse A, de-crosslinked and the bound RNA extracted with Trizol. RNA was polyA-tailed with polyA polymerase *in vitro* and, where indicated, treated with RNase H1. Oligo-dT was used to synthesize cDNA, which was amplified with forward or reverse primers (corresponding to sense and antisense RNA, respectively) and subjected to qRT-PCR. 18S RNA bound to chromatin was used as control to normalize the relative amount of amplified RNA. For details, see Materials and Methods. (**B**) Enlarged map of CAV1 at the E_2_-dependent Start 2 site with the oligonucleotides used to synthesize the RNAs, as shown in (**A**). (**C**) Levels of sense or antisense CAV1 RNAs detected with the specific primers indicated below the histogram. Chromatin-bound RNAs were isolated from cells exposed to E_2_ for 45 min. Where indicated, the chromatin digested with RNAse H1. Fisher Test or ANOVA *p ≤ 0.01 relative to control (−E_2_). The data set corresponding to (**C**) are shown in Supplemental Fig. S6 (**D**) Schematic diagram illustrating the localization of the RNAs detected by oligonucleotides 10 and 7 and the ERE1-polyA site in CAV1 gene ((+) strand, red; (−) strand, green; RNA, blue; RNAse H1, scissors). The blue curved lines represent the RNAs at the 3′ and 5′ of the loop. The yellow circles represent RNA polymerase II localized at these sites following E_2_-induction^10^.

To search for transcripts hybridized to DNA at the CAV15′ and 3′ ends, we isolated sense and antisense RNAs associated with formaldehyde-fixed chromatin before or after E_2_-treatment, and with or without previous RNAse H1 digestion, using 5′ and 3′ primers (Fig. 5A,B and Materials and Methods). Only two primers, located at the 5′ (oligonucleotide 10) and 3′ (oligonucleotide 7) ends of CAV1, detected E_2_-induced antisense RNA. These RNAs were also sensitive to RNase H1 (Fig. 5C and Supplemental Fig. S6). The 5′ antisense RNA (oligonucleotide 10) is located upstream of the E_2_-dependent Start 2, whereas the 3′ antisense RNA localized to the polyA site (Fig. 5B,C, Supplemental Fig. S6). The 5′ and 3′ antisense RNA was significantly induced by E_2_ (see the legend of Fig. 5C and Supplemental Fig. S6 *≤0.001 relative to the control). The genomic segment marked by RNA detected by oligonucleotide 10 and Start 2 is also the main site of accumulation of RNA polymerase II after E_2_ induction (Fig. 5D and ref.^10^). Other sense and antisense RNAs were detected by oligonucleotides spanning the ERE1-Start 2 segment, but none of these transcripts was E_2_-inducible, although all of them were sensitive to RNAse H1 (Fig. 5C).

In conclusion, the genomic segment between ERE1 and Start 2 recruits E_2_ receptor, binds the RNA detected by oligo10 and forms the ERE1-polyA loop upon E_2_ induction (Figs 4 and 5D).

## Discussion

We reported previously the basic structures of CASP9 or BCL2 5′ end loops induced by RA or E_2_, respectively^5,7^. In this report, we applied 3C and other protocols to map precisely their locations and to study their configurations and abundance as a function of time after induction.

We show that there are several configurations of the same intragenic loop that connects the transcription start sites, the RARE or ERE enhancers and the polyA sites of induced genes. We identify two major configurations on the basis of the dependency of the loop stability on RNA-DNA hybrids (R-loops). Treatment of chromatin with RNAse H1 destroys the intragenic loops early after transcription induction, whereas RNAse H1 has no effect on loop stability 60 min after induction. We also demonstrate that loop levels undergo cyclic changes after induction.

We report for the first time that R-loops, as detected by RNAse H1 sensitivity and ChIP probing for RNA:DNA hybrids, can be necessary for loop stability.

**Figure 6:**
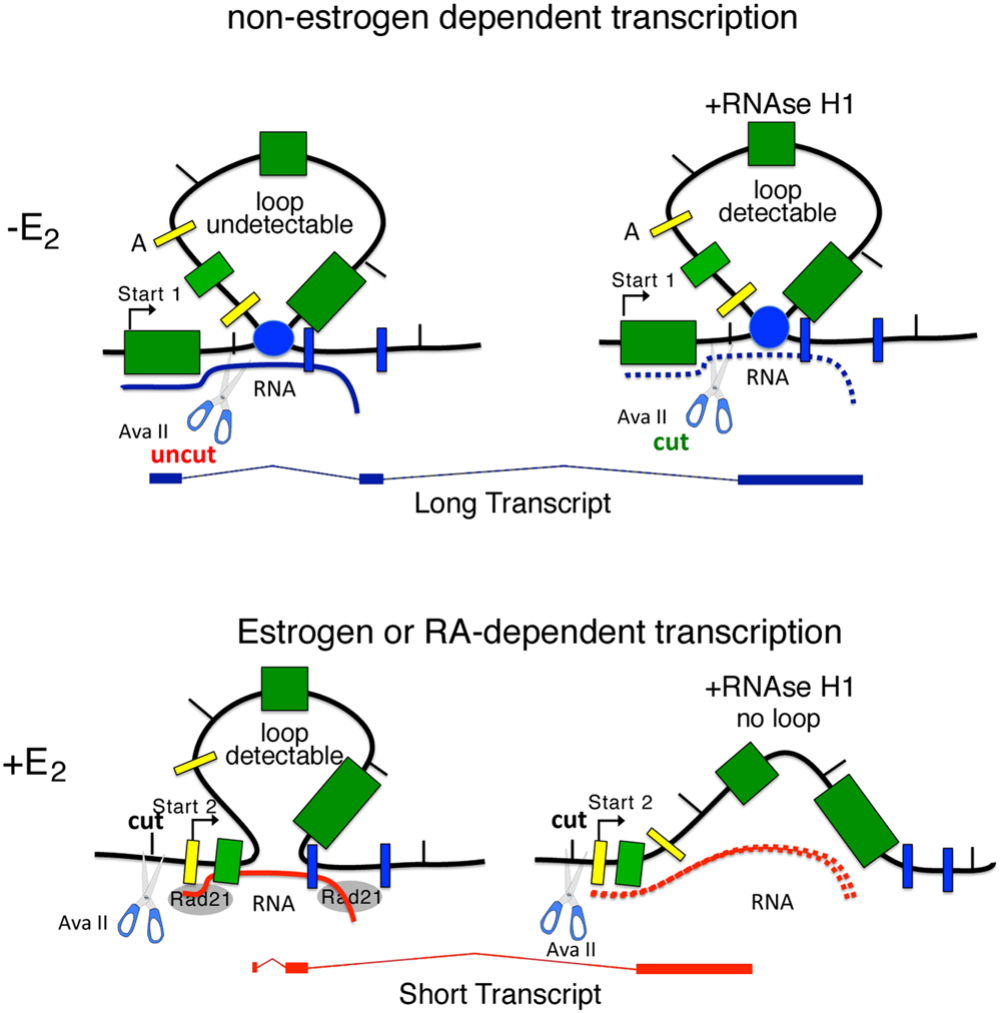
E_2_-induction changes the 5′ end of the CAV1 loop and determines the ends of the short CAV1 transcript. Schematic diagram of the CAV1 promoter-ERE-polyA loops, represented as a single loop, in cells exposed to E_2_. In the absence of E_2_, the loop at the 5′ end (promoter-ERE) was not detectable. The CAV1 transcript synthesized under these conditions is indicated as a long transcript originating from Start 1 (Figs 3A and 4A). Loop modification upon E_2_-induction. E_2_ induces two RNAs corresponding to oligonucleotide 10 and 7 (Fig. 5D) located upstream of Start 2 and at the polyA site, respectively, which conceivably influence this modification. The loop ERE-PolyA, described in Fig. 2D, is now detectable and is sensitive to RNAse H1. The CAV1 transcript synthesized under these conditions is indicated as short transcript originating from Start 2 (Figs 3A and 5).

Loop-stabilizing RNA:DNA hybrids arise during early transcription (15 min after RA-E_2_ addition) preceding mRNA accumulation by 15 min (Fig. 3). At 60 min post-induction, loop stability is no longer sensitive to RNase H1 (Fig. 2B,E). Loops can be detected prior to induction by treatment of the chromatin with RNAse H1. The ribonuclease removes RNA that blocks access of restriction enzyme to the DNA, and hence interferes with 3C analysis (Figs 2B,E, 4C). We identified RNAs complementary to the TSS region and the polyA site of CAV1 gene. These were induced by E_2_ and were sensitive to RNAse H1 (Fig. 5). Co-transcriptional R-loop formation at the TSS during E_2_-induced transcription has been reported and was suggested to be a major cause of DNA damage^11,12^. These RNAs may be responsible for loop stability because they mark the 5′ and 3′ ends of the loop, i.e. the TSS (Start 2) and the polyA site of the E_2_-induced CAV1 transcription unit (Fig. 5B). We suggest that the RNAs may guide RNA Polymerase II to the TSS and polyA sites of the E_2_-induced-transcription unit (Fig. 5B,D). Since CAV1 mRNA induced by E_2_ contains a specific 5′ end (Start 2) contiguous to ERE1, the RNA detected by oligonucleotide 10 marks precisely the 5′ end border of the E_2_-induced transcription unit (Fig. 5B,D). A schematic diagram (Fig. 6) shows the configurations of CAV1 gene loops in the presence or absence of E_2_. The accessibility of the Ava II site upon RNAse H1 treatment reveals that E_2_-stimulation moves the R-loop present in this DNA segment. E_2_-induction shifts the 5′ end of the loops from Start 1 to Start 2, forming the ERE1–3′ intragenic loop (Fig. 2D). This loop is sensitive to RNase H1. At longer times after induction, the loops become resistant to digestion, indicating they are no longer stabilized by RNA:DNA hybrids.

The precise mechanism by which the RNA:DNA hybrids stabilize chromatin loops is not clear. The transcripts corresponding to oligonucleotides 10 and 7 are located precisely at the base of the loop detected by 3C. It is possible that the RNA:DNA hybrid recruits proteins that bind the loop base and stabilize it. We show that RAD21, the cohesin subunit that is also involved in DNA repair, is recruited at the promoter, enhancer and polyA sites of CAV1. The timing of the recruitment is identical to the formation of the 5′-3′ ends loop. Furthermore, RNAse H1 treatment prevents RAD21 binding to chromatin (Figs 1D, 4D and Supplemental Fig. S3), suggesting that the RNA-DNA hybrids at the 5′ and 3′ ends also stabilize cohesin binding. Recruitment of DNA double-strand break repair factors by specific RNA:DNA hybrids has, in fact, been reported^13^.

Changes in loop configuration depends upon epigenetic chromatin modification, including changes in the histone code, as well as repair enzyme recruitment at promoter and polyA. Inhibition of the histone demethylase LSD1 or NER early after hormonal induction inhibited both transcription and loop formation^5,7,14^.

These RNA-dependent loops represent lower-scale chromatin structures associated with gene-specific transcription, compared to TAD and other longer-range chromatin-chromosome contacts.

We propose that the RNA-stabilized loops mark the borders of the transcription units and may define the precise 5′ start site(s) following induction of transcription by various effectors.

## Materials and Methods

### Cells and transfections

Human breast cancer MCF-7 cells were grown at 37 °C in 5% CO_2_ in Dulbecco’s modified Eagle’s medium (DMEM) supplemented with phenol red, L-glutamine (2 mM), insulin (10 μg/ml), hydrocortisone (3.75 ng/ml) and 10% fetal bovine serum (FBS) (Invitrogen). To evaluate the effect of Retinoic Acid (RA) or Estrogen (E_2_), MCF-7 cells were grown in phenol red-free medium containing 10% dextran-charcoal-stripped FBS for 1–3 days, before being challenged with 300 nM RA or 50 nM E2 for different times according to the experimental protocol.

### Chromosome conformation capture

Following E_2_ or RA treatment, MCF-7 cells (2.5 × 10^6^) were cross-linked in 12 ml of PBS with 1% formaldehyde for 10 min at room temperature. The reaction was quenched by addition of glycine to a final concentration of 125 mM. Fixed cells were harvested and the pellet resuspended in 1 ml of ice-cold lysis buffer (10 mM Tris-HCl pH 8.0, 10 mM NaCl, 0.2% NP40). Nuclei were washed with 0.5 ml of restriction enzyme buffer, centrifuged and resuspended in 100 μl of restriction enzyme buffer. Sodium dodecyl sulphate (SDS) was added to a final concentration of 0.1%, and nuclei were incubated at 37 °C for 15 min. Triton X-100 was added to a final concentration of 1% to sequester SDS. Chromatin from MCF-7 cells was digested with NcoI, Bam HI, and Ava II restriction enzymes for CASP9, BCL2 and CAV1 3C analysis, respectively.

Digestion was performed with 100 U of restriction enzyme at 37 °C for 16 h. Where indicated, digestion with RNase H1 (100 U/ml) of fixed chromatin for 60 min was carried out before ligation. The 3C protocol used is described in ref.^5^. Restriction enzymes were inactivated by addition of SDS to 2% and incubation at 65 °C for 30 min. The reaction mix was diluted into 1 ml ligation reaction buffer and incubated at 16 °C for 18 h with 50 U of T4 DNA Ligase (Roche Applied Science). EDTA (10 mM) was added to stop the reactions. Samples were treated with Proteinase K (200 μg/ml) and incubated for 5 h at 55 °C and then overnight at 65 °C to reverse formaldehyde crosslinks. DNA was then purified by phenol/chloroform extraction and ethanol precipitation. Samples were redissolved in 20 μl of Tris-EDTA (TE) buffer. To prepare a control template, we used a pool of plasmids containing an equimolar amount of inserts spanning the genomic regions of interest. Five μg of plasmid DNA were digested with each restriction enzyme in 50 μl of 1x buffer for 8 h at 37 °C and then ligated in 20 μl with 5 U of T4 ligase at 16 °C for 4 h. The efficiency of digestion at the end of 3C treatment was quantified by real-time PCR, amplifying a fragment spanning two digestion sites (uncut) in different 3C DNA preparations. Primer sequences are described in Supplemental Table S1. PCR products were separated on 1.2% agarose gels, stained with ethidium bromide and quantified with the imageJ program (Rasband WS, ImageJ, National Institutes of Health, Bethesda, MD, USA; http://rsb.info.nih.gov/ij/). The specific amplified fragments at the end of the procedure were verified by DNA sequence analysis. For details see ref.^5^.

### DRIP S9.6

Following E_2_ or RA treatment, MCF-7 cells (2.5 × 10^6^) genomic DNA was extracted and 10 µg were digested with restriction enzymes (30 U in total, 10 U BamHI, Hind III, and KpnI for CASP9, BamHI and HaeIII for CAV1 and BamHI for BCL2). Digestion was performed at 37 °C for 16 h in the presence of 50 µg/ml of RNAse A. The samples were split in two aliquots; one was digested with 20U of RNAse H1 (5U/ul) for 60 minutes at 37 °C. The DNA was extracted with 1 volume of phenol/chloroform/isoamyl alcohol (25:24:1) and precipitated in LiCl 0.4 M/ethanol 75%. Samples were redissolved in 15 µl of TE buffer (10 mM Tris-HCl, 1 mM EDTA, pH 8,0). 4 µg of digested DNA was diluted in 300 µl of TE buffer, in the presence of 10X Binding Buffer (100 mM NaPO_4_ pH 7.0, 1.4 M NaCl, 0.5% Triton X-100) and 2 µg of S9.6 antibody (Kerafast, ENH001). Samples were incubated at 4 °C for 16 h on a rotaryshaker. The immuno-complexes were recovered by incubation for 2 h at 4 °C with 40 μl of protein-A/G PLUS agarose, beads were washed with binding buffer 1X for four times. Beads were suspended in 250 µl of Elution Buffer (50 mM Tris-HCl pH 8.0, 10 mM EDTA, 1% SDS) and incubated with Proteinase K (150 µg/sample) for 2 h at 55 °C. Immunoprecipitated DNA was recovered by phenol/chloroform/isoamyl alcohol extraction and ethanol precipitation and redissolved in TE buffer. Samples were subjected to qPCR using the primers reported in Supplemental Table 1. Real Time-qPCRs were performed using FastStart Universal SYBR Green Master (Rox) (Roche Applied Science).

#### Chromatin Immuno-Precipitation (ChIP)

Cells were treated as indicated in the legends to the figures. The cells (~2.5 × 10^6^ for each antibody) were harvested and nuclei isolated, treated or not treated with 20 U of RNAse H1 (5 U/ul) for 30 minutes at 37 °C and fixed for 10 min at room temperature by adding 1 volume of 2% formaldehyde to a final concentration of 1%; the reaction was quenched by addition of glycine to a final concentration of 125 mM. Fixed cells were harvested and the pellet was resuspended in 1 ml of Lysis Buffer (10 mM Tris-HCl pH 8.0, 10 mM NaCl, 0.2% NP40) containing 1X protease inhibitor cocktail (Roche Applied Science). The lysates were digested with Micrococcal Nuclease (Cell Signaling #10011) 100 U/sample in the presence of 10X Digestion Buffer (10%Triton X-100; 50 mM CaCl_2_, 1.5 M NaCl, 0.5 M Tris-HCl pH 8.0) for 10 minutes at 37 °C and then subjected to 3 rounds of 20-second pulses. The nuclease was inactivated by addition of 50 mM EDTA and incubation at room temperature for 5 min. Digested samples were centrifuged and an aliquot (1/10) of supernatants was further treated with Proteinase K (200 µg/ml) extracted with 1 volume of phenol/chloroform/isoamyl alcohol (25:24:1) and precipitated in LiCl 0.4 M/ethanol 75% to determine DNA concentration and shearing efficiency (input DNA). 200 µl of Dynabeads Protein A/G was washed suspended in the same volume of PBS/0.01% Tween in presence of 4 µg of antibody Rad21 (sc-166973) (1 µg/sample). The sheared chromatin was incubated with 50 µl of Dynabeads Protein A/G (Invitrogen) antibody coniugated at 4 °C for 2 h on a rotary shaker. The beads were washed with wash buffers according to the manufacturer’s instructions. Samples were treated with Proteinase K (100 µg/ml) and incubated for 2 h at 55 °C and then overnight at 65 °C to reverse formaldehyde cross-links. The immunoprecipitated DNA was recovered by phenol/chloroform/isoamyl alcohol extraction and ethanol precipitation and redissolved in TE buffer (10 mM Tris-HCl, 1 mM EDTA, pH 8.0). Samples were subjected to qPCR using the primers reported in Supplementary Table 1. Real Time-qPCRs were performed using FastStart Universal SYBR Green Master (Rox) (Roche Applied Science).

### RNA extraction, qPCR and qRT-PCR

Total RNA was extracted using TRIzol (Gibco/Invitrogen). cDNA was synthesized in 20-μl reactions containing 1 μg of total RNA, 100 U of Superscript III Reverse Transcriptase (Invitrogen) and 2 μl random hexamer (20 ng/μl) (Invitrogen). mRNA was reverse-transcribed for 1 h at 50 °C; the reaction was then heat-inactivated for 15 min at 70 °C. The products were stored at −20 °C. qRT-PCR and qPCR were performed on a 7500 Real Time PCR System (Applied Biosystems) using the SYBR Green-detection system (FS Universal SYBR Green Master Rox/Roche Applied Science). The complete list of oligonucleotides used is reported in Supplementary Table.

### Sense and antisense nascent mRNA isolation

An aliquot of formaldehyde–fixed chromatin, treated or not treated with RNase H1, used for 3C, from E_2_-exposed and control cells, was de-cross-linked and the bound RNA was extracted with Trizol. A polyA tail was then added to purified RNA with polyA polymerase *in vitro*. The Oligo-dT was used to synthesize cDNA, which was amplified with forward or reverse primers (corresponding to sense and antisense RNA, respectively) and subjected to qRT-PCR. 18S RNA bound to chromatin was used as control to normalize the relative amount of amplified RNA. A schematic representation of the method is shown in Fig. 5A.

### Statistical analysis

All data are presented as mean ± standard deviation in at least three experiments in triplicate (n 9). Data sets were analyzed statistically using JMP Statistical Discovery™ software by SAS and tested for normality using the Shapiro-Wilks test (“normal distribution fit” tool -JMP software). Two-tailed significance tests were performed with p < 0.05 considered significant. Non-parametric analysis was performed with the Mann-Whitney-U-Test (Wilcoxon rank-sum test), parametric with the t-test. Detailed statistical analysis of the data is reported in Supplemental Statistical Figs 1–5.

## Supplementary information

Supplementary Data
