# Supplementary material for: RNA Stabilizes Transcription-Dependent Chromatin Loops Induced By Nuclear Hormones
Source: Sci Rep. 2019 Mar 8;9:3925. doi: 10.1038/s41598-019-40123-6 (PMC6408484; doi:10.1038/s41598-019-40123-6)

Supplementary data for

RNA stabilizes transcription-dependent chromatin loops induced by nuclear hormones

Antonio Pezone[1*,] Candida Zuchegna[2*], Alfonso Tramontano[1], Antonella Romano[2], Giusi Russo[1], Mariarosaria de Rosa[1], Maria Vinciguerra[3], Antonio Porcellini[2]‡, Max E. Gottesman[4] and Enrico V. Avvedimento[1] ‡

1. Dipartimento di Medicina Molecolare e Biotecnologie Mediche, Università Federico II Napoli, Italy
2. Dipartimento di Biologia, Università Federico II Napoli, Italy
3. DNA Metabolism Laboratory, IFOM, The FIRC Institute of Molecular Oncology, Milan, Italy, .
3. Institute of Cancer Research, Columbia University Medical Center, New York, NY, 10032, USA.
** These authors contributed equally to this work.*
‡ Correspondence and requests for materials should be addressed to M.G. (email: meg8@columbia.edu), A.P. (email: antonio.porcellini@unina.it) or E.V.A. (email: avvedim@unina.it)

Supplement Index:

Supplementary Table S1 page 2
Supplementary Figures legends pages 3-4
Supplementary Figures S1-S6 pages 5-10
Original Gels displayed in Fig 1 page 11
Original Gels displayed in Fig 2 page 12

Supplementary Table S1

CASP9-1	5′-GTCTGTACATGTTCAGTACAATGC-3′	*CASP9*
CASP9-2	5′-CCACCTGCAGCTCTTCCA-3′	*CASP9*
CASP9-3	5′-CAGTCATCCGGAGACCTAAACC-3′	*CASP9*
CASP9-4	5′-AGATGCCCAGCACTATGCTAAG-3′	*CASP9*
CASP9-5	5′-GGTGCCTGGCAAATAGCAAT-3′	*CASP9*
CASP9-6	5′-TCAACAAAAATTCACCAAAACTCA-3′	*CASP9*
CASP9-7	5′-GCAAGTACTCAATAATGTTCACC-3′	*CASP9*
CASP9-8	5′-GGGGTGATTCCAGCATAGGTTC-3′	*CASP9*
CASP9-9	5′-CTTCTGCGTCTGAACTTGAACC-3′	*CASP9*
CASP9-10	5′-CTGAAAGAAAGAAGGCTGGATGC-3′	*CASP9*
CASP9-11	5′-AGGAAGGAAACTACCGCTTGCT-3′	*CASP9*
CASP9-12	5′-TCCCTTTCACCGAAACAGCA-3′	*CASP9*
BCL2-1	5′-ATCACAGGACTTCTGCGAATACCG-3′	*BCL2*
BCL2-2	5′-GTGGAGCCGGCGAAATAAAATC-3′	*BCL2*
BCL2-3	5′-TGCAGCCCAGACAAATGTGGTTAC-3′	*BCL2*
BCL2-4	5′-CTCCCTGATCCAAACTTGGGAATG-3′	*BCL2*
BCL2-5	5′-TATGCGCGTGGGAGGTGT-3′	*BCL2*
BCL2-6	5′-GGACGGGGTGAACTGGGGGAGGAT-3′	*BCL2*
BCL2-7	5′-TCTGCCTTCCCCATTCAACTATTA-3′	*BCL2*
BCL2-8	5′-CCCCTTCCTCTTGCTGGTATC-3′	*BCL2*
BCL2-9	5′-GATAGTGATGAAGTACATCC-3′	*BCL2*
BCL2-10	5′-GAGGGTCAGGTGGACCACAGGTG-3′	*BCL2*
BCL2-RNAF	5′-GTGGTGGAGGAGCTCTTCAG-3′	*BCL2*
BCL2-RNAR	5′- CAAACTGAGCAGAGTCTTCAG-3′	*BCL2*
BCL2- RNAR2	5′-CCAAGTGCACCTACCCAGCC-3′	*BCL2*
BCL2- PolyAF*	5′- AGATACTGGTTGTGCCTGGAATTGT-3′	*BCL2*
BCL2- PolyAR*	5′- GTAAGGCAATCCCTTAGCCTGGTCCCT-3′	*BCL2*
BCL2- EREF*	5′- GTCGCCAGGACCTCGCCGCTGCA-3′	*BCL2*
BCL2- ERER*	5′- CTGGCTGGACATCTCGGCGAAGT-3′	*BCL2*
CAV1-1	5′-GAAGAGAAGCCAGGAATGTTTTAT-3′	*CAV1*
CAV1-2	5′-CTGCTGGGGGTTCGAAGAGGTG-3′	*CAV1*
CAV1-3	5′-CCGGGAGAAGCCTGCGGCTGC-3′	*CAV1*
CAV1-4	5′-CCTTGGGACGGTGAGATG-3′	*CAV1*
CAV1-5	5′-CGTATTTTCTTTGTCTTTAGTCCT-3′	*CAV1*
CAV1-6	5′-TGCCTCCTCCCCCATCTT-3′	*CAV1*
CAV1-7	5′-CACAGACTCTGACACATAAACACCTG-3′	*CAV1*
CAV1-8	5′-AAGTTGGGTGTGAGTGGATTTAAAG-3′	*CAV1*
CAV1-9	5′-GGATCTTAGATAAAGCTGGAAGG-3′	*CAV1*
CAV1-10	5′-CTAAGCCGAGTCGCTCTCGC-3′	*CAV1*
CAV1-11	5′- CAGGACCCGCAGGACCTCGGC-3′	*CAV1*
CAV1-12	5′-CTGCAGGGCGCGCCTGCCTC-3′	*CAV1*
CAV1-13	5′-GATCTCGCAGAGGACACCACAC-3′	*CAV1*
CAV1-14	5′-AGGACTAAAGACAAAGAAAATACG-3′	*CAV1*
CAV1-15/ERE3R*	5′-GAACTCCAACCTGAATGTGC-3′	*CAV1*
CAV1-ERE1F*	5′-TAAAGCTGGAAGGGATTACCGG-3′	*CAV1*
CAV1-ERE1R*	5′-CTTCTCCCGGACTCCCTAAG-3′	*CAV1*
CAV1-ERE2F*	5′-ACCCTAAACACCTCAACGAT-3′	*CAV1*
CAV1-ERE2R*	5′-CTGCCAGAGGAGAGCTGTCCCAG-3′	*CAV1*
CAV1-ERE3F*	5′-GGACACCTGCACTTAGATGT-3′	*CAV1*
CAV1-PolyAF*	5′- AGACATGTCTGTTCTACATAGATGCT-3′	*CAV1*
CAV1-PolyAR*	5′- CAGGCTTGTAACCTTTACAGGACATGCAT-3′	*CAV1*
CAV1-IntronF*	5′- AGGCACGGAGCACGTCATGA-3′	*CAV1*
CAV1-IntronRF*	5′- GCTTTGATGAATGCCACAGCAGAG-3′	*CAV1*
CAV1-RNAF	5′-GCCGCCCTCCCCGTCCTG-3′	*CAV1*
CAV1-RNAF	5′-GCCGCCCTCCCCGTCCTG-3′	*CAV1*
CAV1-RNAFB	5′-GGCCAACCGCGAGCAGAACAAA-3′	*CAV1*
CAV1-RNAR	5′-GAGGGCAGACAGCAAGCGGTAAAA-3	*CAV1*
18S-1	5′-GCGCTACACTGACTGGCTC-3′	*18S*
18S-2	5′-CATCCAATCGGTAGTAGCGAC-3′	*18S*
ControlF	5′- ACCGAGACCCCTCTTGCTCT -3′	*TSHR*
ControlR	5′- AGTTGCTAACAGTGATGAGAGGCT -3′	*TSHR*

Supplementary Table S1. Complete list of DNA oligonucleotides used for PCR. On the left is shown the primer identification tag (ID); on the centre, the DNA sequence; on the right, the genes or loci corresponding to the specific primers.

Supplementary Figures

Suppl. Figure 1S

Statistical analysis of the data presented in Fig. 1C (A) and 1D (B).

The diamond illustrates a sample mean and 95% confidence intervals, the mean line across the middle of each diamond represents the group mean, the horizontal size of the diamond is proportional to the sample size. Differences between groups were tested by ANOVA.

Suppl. Figure 2S

Statistical analysis of the data presented in Fig. 2C (A) and 2D (B).

The diamond illustrates a sample mean and 95% confidence intervals, the mean line across the middle of each diamond represents the group mean, the horizontal size of the diamond is proportional to the sample size. Differences between groups were tested by ANOVA.

Suppl. Figure 3S

RAD21 binding to the BCL2 promoter-ERE and PolyA sites.

ChIP was carried out as described in Materials and Methods, including the antibody used and RNAse H1 digestion. The data set corresponding are shown.

Suppl. Figure 4S

Statistical analysis of the data presented in Fig. 2E (A) and 2F (B).

The diamond illustrates a sample mean and 95% confidence intervals, the mean line across the middle of each diamond represents the group mean, the horizontal size of the diamond is proportional to the sample size. Differences between groups were tested by ANOVA.

Suppl. Figure 5S

Statistical analysis of the data presented in Fig. 4B (A) and 4D (B).

The diamond illustrates a sample mean and 95% confidence intervals, the mean line across the middle of each diamond represents the group mean, the

horizontal size of the diamond is proportional to the sample size. Differences between groups were tested by ANOVA.

Suppl. Figure 6S

Statistical analysis of the data presented in Fig. 5C.

The diamond illustrates a sample mean and 95% confidence intervals, the mean line across the middle of each diamond represents the group mean, the horizontal size of the diamond is proportional to the sample size. Differences between groups were tested by ANOVA.

Supplementary Figure 1

A

Oneway Analysis of RA By Time PCR=Promoter

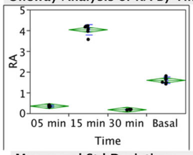

Means and Std Deviations

Level	Number	Mean	Std Dev	Std Err Mean
05 min	6	0.34617	0.042939	0.01753
15 min	6	4.02850	0.246996	0.10084
30 min	6	0.17200	0.015723	0.00642
Basal	6	1.59083	0.146046	0.05962

Means Comparisons

Comparisons for each pair using Student's t

Level	- Level	Difference	Lower CL	Upper CL	p-Value
15 min	30 min	3.856500	3.68153	4.031468	<.0001*
15 min	05 min	3.682333	3.50737	3.857301	<.0001*
15 min	Basal	2.437667	2.26270	2.612634	<.0001*
Basal	30 min	1.418833	1.24387	1.593801	<.0001*
Basal	05 min	1.244667	1.06970	1.419634	<.0001*
05 min	30 min	0.174167	-0.00080	0.349134	0.0510

Oneway Analysis of RA+RNAse H1 By Time PCR=Promoter

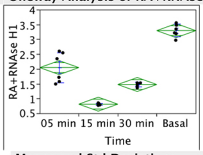

Matched Pairs 15 min

Wilcoxon Sign-Rank

RA+RNAse H1-RA

Test Statistic	-10.500*
Prob > |z|	0.0313*
Prob > z	0.9844
Prob < z	0.0156*

Means and Std Deviations

Level	Number	Mean	Std Dev	Std Err Mean
05 min	6	2.04317	0.506271	0.20668
15 min	6	0.80967	0.029891	0.01220
30 min	6	1.47267	0.068736	0.02806
Basal	6	3.29150	0.212895	0.08691

Means Comparisons

Comparisons for each pair using Student's t

Level	- Level	Difference	Lower CL	Upper CL	p-Value
Basal	15 min	2.481833	2.148051	2.815616	<.0001*
Basal	30 min	1.818833	1.485051	2.152616	<.0001*
Basal	05 min	1.248333	0.914551	1.582116	<.0001*
05 min	15 min	1.233500	0.899717	1.567283	<.0001*
30 min	15 min	0.663000	0.329217	0.996783	0.0005*
05 min	30 min	0.570500	0.236717	0.904283	0.0019*

Oneway Analysis of RA By Time PCR=RARE

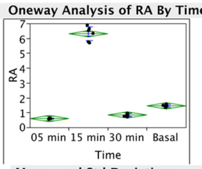

Means and Std Deviations

Level	Number	Mean	Std Dev	Std Err Mean
05 min	6	0.59283	0.030433	0.01242
15 min	6	6.30067	0.482393	0.19694
30 min	6	0.83500	0.099122	0.04047
Basal	6	1.44817	0.081935	0.03345

Means Comparisons

Comparisons for each pair using Student's t

Level	- Level	Difference	Lower CL	Upper CL	p-Value
15 min	05 min	5.707833	5.40665	6.009017	<.0001*
15 min	30 min	5.465667	5.16448	5.766850	<.0001*
15 min	Basal	4.852500	4.55132	5.153684	<.0001*
Basal	05 min	0.855333	0.55415	1.156517	<.0001*
Basal	30 min	0.613167	0.31198	0.914350	0.0004*
30 min	05 min	0.242167	-0.05902	0.543350	0.1091

Oneway Analysis of RA+RNAse H1 By Time PCR=RARE

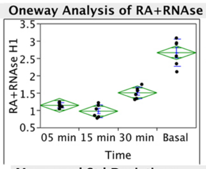

Matched Pairs 15 min

Wilcoxon Sign-Rank

RA+RNAse H1-RA

Test Statistic	-10.500*
Prob > |z|	0.0313*
Prob > z	0.9844
Prob < z	0.0156*

Means and Std Deviations

Level	Number	Mean	Std Dev	Std Err Mean
05 min	6	1.13983	0.069620	0.02842
15 min	6	0.97033	0.186099	0.07597
30 min	6	1.50183	0.167738	0.06848
Basal	6	2.65800	0.383749	0.15666

Means Comparisons

Comparisons for each pair using Student's t

Level	- Level	Difference	Lower CL	Upper CL	p-Value
Basal	15 min	1.687667	1.40853	1.966801	<.0001*
Basal	05 min	1.518167	1.23903	1.797301	<.0001*
Basal	30 min	1.156167	0.87703	1.435301	<.0001*
30 min	15 min	0.531500	0.25237	0.810634	0.0008*
30 min	05 min	0.362000	0.08287	0.641134	0.0136*
05 min	15 min	0.169500	-0.10963	0.448634	0.2198

Oneway Analysis of RA By Time PCR=PolyA

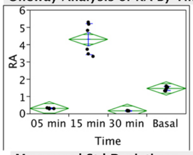

Means and Std Deviations

Level	Number	Mean	Std Dev	Std Err Mean
05 min	6	0.29717	0.020024	0.00817
15 min	6	4.29133	0.897916	0.36657
30 min	6	0.15867	0.010250	0.00418
Basal	6	1.45167	0.125438	0.05121

Means Comparisons

Comparisons for each pair using Student's t

Level	- Level	Difference	Lower CL	Upper CL	p-Value
15 min	30 min	4.132667	3.58655	4.678779	<.0001*
15 min	05 min	3.994167	3.44805	4.540279	<.0001*
15 min	Basal	2.839667	2.29355	3.385779	<.0001*
Basal	30 min	1.293000	0.74689	1.839113	<.0001*
Basal	05 min	1.154500	0.60839	1.700613	0.0003*
05 min	30 min	0.138500	-0.40761	0.684613	0.6026

Oneway Analysis of RA+RNAse H1 By Time PCR=PolyA

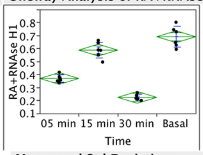

Matched Pairs 15 min

Wilcoxon Sign-Rank

RA+RNAse H1-RA

Test Statistic	-10.500*
Prob > |z|	0.0313*
Prob > z	0.9844
Prob < z	0.0156*

Means and Std Deviations

Level	Number	Mean	Std Dev	Std Err Mean
05 min	6	0.369500	0.028183	0.01151
15 min	6	0.587500	0.060504	0.02470
30 min	6	0.224000	0.021531	0.00879
Basal	6	0.690500	0.080261	0.03277

Means Comparisons

Comparisons for each pair using Student's t

Level	- Level	Difference	Lower CL	Upper CL	p-Value
Basal	30 min	0.4665000	0.4023178	0.5306822	<.0001*
15 min	30 min	0.3635000	0.2993178	0.4276822	<.0001*
Basal	05 min	0.3210000	0.2568178	0.3851822	<.0001*
15 min	05 min	0.2180000	0.1538178	0.2821822	<.0001*
05 min	30 min	0.1455000	0.0813178	0.2096822	0.0001*
Basal	15 min	0.1030000	0.0388178	0.1671822	0.0032*

Oneway Analysis of RA By Time PCR=Control

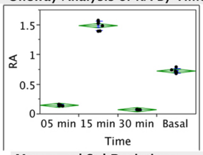

Means and Std Deviations

Level	Number	Mean	Std Dev	Std Err Mean
05 min	6	0.14100	0.010237	0.00418
15 min	6	1.48050	0.077058	0.03146
30 min	6	0.06633	0.003615	0.00148
Basal	6	0.71717	0.039367	0.01607

Means Comparisons

Comparisons for each pair using Student's t

Level	- Level	Difference	Lower CL	Upper CL	p-Value
15 min	30 min	1.414167	1.361652	1.466681	<.0001*
15 min	05 min	1.339500	1.286985	1.392015	<.0001*
15 min	Basal	0.763333	0.710819	0.815848	<.0001*
Basal	30 min	0.650833	0.598319	0.703348	<.0001*
Basal	05 min	0.576167	0.523652	0.628681	<.0001*
05 min	30 min	0.074667	0.022152	0.127181	0.0076*

Oneway Analysis of RA+RNAse H1 By Time PCR=Control

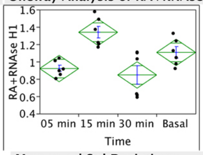

Matched Pairs 15 min

Wilcoxon Sign-Rank

RA+RNAse H1-RA

Test Statistic	-9.5000*
Prob > |z|	0.0625
Prob > z	0.9688
Prob < z	0.0313*

Means and Std Deviations

Level	Number	Mean	Std Dev	Std Err Mean
05 min	6	0.92050	0.091005	0.03715
15 min	6	1.33867	0.168139	0.06864
30 min	6	0.84633	0.259158	0.10580
Basal	6	1.10633	0.156105	0.06373

Means Comparisons

Comparisons for each pair using Student's t

Level	- Level	Difference	Lower CL	Upper CL	p-Value
15 min	30 min	0.4923333	0.276825	0.7078413	0.0001*
15 min	05 min	0.4181667	0.202659	0.6336746	0.0006*
Basal	30 min	0.2600000	0.044492	0.4755080	0.0205*
15 min	Basal	0.2323333	0.016825	0.4478413	0.0360*
Basal	05 min	0.1858333	-0.029675	0.4013413	0.0872
05 min	30 min	0.0741667	-0.141341	0.2896746	0.4811

B

Oneway Analysis of RA By Time PCR=RARE

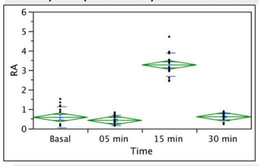

Means and Std Deviations

Level	Number	Mean	Std Dev	Lower 95%	Upper 95%
Basal	15	0.57892	0.522615	0.2895	0.8683
05 min	15	0.42177	0.248711	0.2840	0.5595
15 min	15	3.26995	0.603719	2.9356	3.6043
30 min	15	0.60659	0.205633	0.4927	0.7205

Means Comparisons

Comparisons for each pair using Student's t

Level	- Level	Difference	Lower CL	Upper CL	p-Value
15 min	05 min	2.848177	2.53318	3.163169	<.0001*
15 min	Basal	2.691028	2.37604	3.006020	<.0001*
15 min	30 min	2.663355	2.34836	2.978347	<.0001*
30 min	05 min	0.184822	-0.13017	0.499814	0.2448
Basal	05 min	0.157149	-0.15784	0.472141	0.3219
30 min	Basal	0.027673	-0.28732	0.342665	0.8609

Oneway Analysis of RA+RNAse H1 By Time PCR=RARE

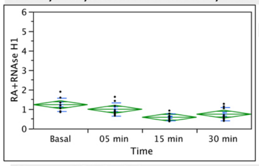

Matched Pairs 15 min

Wilcoxon Sign-Rank

RA+RNAse H1-RA

Test Statistic	-22.500
Prob > |z|	0.0039*
Prob > z	0.9980
Prob < z	0.0020*

Means and Std Deviations

Level	Number	Mean	Std Dev	Lower 95%	Upper 95%
Basal	9	1.22823	0.338007	0.96841	1.4880
05 min	9	0.99402	0.338579	0.73377	1.2543
15 min	9	0.58238	0.178920	0.44485	0.7199
30 min	9	0.74047	0.323552	0.49177	0.9892

Means Comparisons

Comparisons for each pair using Student's t

Level	- Level	Difference	Lower CL	Upper CL	p-Value
Basal	15 min	0.6458492	0.355558	0.9361404	<.0001*
Basal	30 min	0.4877549	0.197464	0.7780461	0.0017*
05 min	15 min	0.4116424	0.121351	0.7019336	0.0069*
05 min	30 min	0.2535481	-0.036743	0.5438392	0.0847
Basal	05 min	0.2342068	-0.056084	0.5244980	0.1101
30 min	15 min	0.1580943	-0.132197	0.4483855	0.2756

Oneway Analysis of RA By Time PCR=Promoter

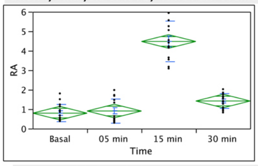

Means and Std Deviations

Level	Number	Mean	Std Dev	Lower 95%	Upper 95%
Basal	15	0.79667	0.44645	0.5494	1.0439
05 min	15	0.90564	0.62126	0.5616	1.2497
15 min	15	4.47673	1.04241	3.8995	5.0540
30 min	15	1.41986	0.37866	1.2102	1.6296

Means Comparisons

Comparisons for each pair using Student's t

Level	- Level	Difference	Lower CL	Upper CL	p-Value
15 min	Basal	3.680060	3.18729	4.172829	<.0001*
15 min	05 min	3.571083	3.07831	4.063853	<.0001*
15 min	30 min	3.056866	2.56410	3.549636	<.0001*
30 min	Basal	0.623193	0.13042	1.115963	0.0141*
30 min	05 min	0.514217	0.02145	1.006986	0.0411*
05 min	Basal	0.108977	-0.38379	0.601746	0.6595

Oneway Analysis of RA+RNAse H1 By Time PCR=Promoter

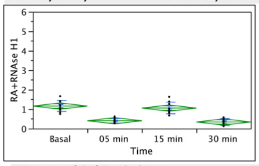

Matched Pairs 15 min

Wilcoxon Sign-Rank

RA+RNAse H1-RA

Test Statistic	-22.500
Prob > |z|	0.0039*
Prob > z	0.9980
Prob < z	0.0020*

Means and Std Deviations

Level	Number	Mean	Std Dev	Lower 95%	Upper 95%
Basal	9	1.15988	0.296262	0.93215	1.3876
05 min	9	0.40593	0.120843	0.31305	0.4988
15 min	9	1.05542	0.312412	0.81528	1.2956
30 min	9	0.34332	0.166948	0.21499	0.4716

Means Comparisons

Comparisons for each pair using Student's t

Level	- Level	Difference	Lower CL	Upper CL	p-Value
Basal	30 min	0.8165580	0.587386	1.045731	<.0001*
Basal	05 min	0.7539418	0.524769	0.983114	<.0001*
15 min	30 min	0.7121059	0.482933	0.941278	<.0001*
15 min	05 min	0.6494897	0.420317	0.878662	<.0001*
Basal	15 min	0.1044522	-0.124720	0.333625	0.3602
05 min	30 min	0.0626162	-0.166556	0.291789	0.5817

Oneway Analysis of RA By Time PCR=PolyA

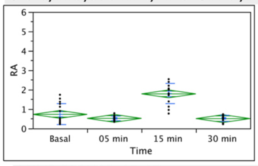

Means and Std Deviations

Level	Number	Mean	Std Dev	Lower 95%	Upper 95%
Basal	15	0.72530	0.541467	0.4254	1.0252
05 min	15	0.52101	0.117443	0.4560	0.5860
15 min	15	1.78244	0.519905	1.4945	2.0703
30 min	15	0.50942	0.158247	0.4218	0.5971

Means Comparisons

Comparisons for each pair using Student's t

Level	- Level	Difference	Lower CL	Upper CL	p-Value
15 min	30 min	1.273013	0.989164	1.556861	<.0001*
15 min	05 min	1.261429	0.977580	1.545278	<.0001*
15 min	Basal	1.057138	0.773289	1.340987	<.0001*
Basal	30 min	0.215875	-0.067974	0.499724	0.1333
Basal	05 min	0.204291	-0.079558	0.488140	0.1549
05 min	30 min	0.011584	-0.272265	0.295433	0.9351

Oneway Analysis of RA+RNAse H1 By Time PCR=PolyA

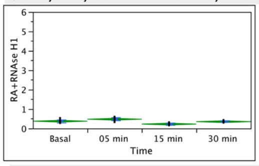

Matched Pairs 15 min

Wilcoxon Sign-Rank

RA+RNAse H1-RA

Test Statistic	-22.500
Prob > |z|	0.0039*
Prob > z	0.9980
Prob < z	0.0020*

Means and Std Deviations

Level	Number	Mean	Std Dev	Lower 95%	Upper 95%
Basal	9	0.375773	0.083324	0.31172	0.43982
05 min	9	0.478063	0.088373	0.41013	0.54599
15 min	9	0.226423	0.052361	0.18617	0.26667
30 min	9	0.351584	0.047220	0.31529	0.38788

Means Comparisons

Comparisons for each pair using Student's t

Level	- Level	Difference	Lower CL	Upper CL	p-Value
05 min	15 min	0.2516400	0.184212	0.3190678	<.0001*
Basal	15 min	0.1493497	0.081922	0.2167775	<.0001*
05 min	30 min	0.1264788	0.059051	0.1939067	0.0006*
30 min	15 min	0.1251612	0.057733	0.1925890	0.0006*
05 min	Basal	0.1022903	0.034862	0.1697181	0.0041*
Basal	30 min	0.0241885	-0.043239	0.0916164	0.4703

Oneway Analysis of RA By Time PCR=Control

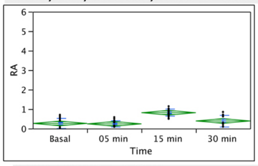

Means and Std Deviations

Level	Number	Mean	Std Dev	Lower 95%	Upper 95%
Basal	15	0.263093	0.261291	0.11839	0.40779
05 min	15	0.241144	0.170355	0.14680	0.33548
15 min	15	0.813595	0.193217	0.70659	0.92059
30 min	15	0.389583	0.302363	0.22214	0.55703

Means Comparisons

Comparisons for each pair using Student's t

Level	- Level	Difference	Lower CL	Upper CL	p-Value
15 min	05 min	0.5724514	0.398561	0.7463416	<.0001*
15 min	Basal	0.5505018	0.376612	0.7243919	<.0001*
15 min	30 min	0.4240124	0.250122	0.5979026	<.0001*
30 min	05 min	0.1484390	-0.025451	0.3223292	0.0928
30 min	Basal	0.1264894	-0.047401	0.3003796	0.1507
Basal	05 min	0.0219496	-0.151941	0.1958398	0.8013

Oneway Analysis of RA+RNAse H1 By Time PCR=Control

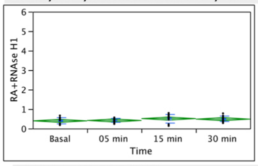

Means and Std Deviations

Level	Number	Mean	Std Dev	Lower 95%	Upper 95%
Basal	15	0.395024	0.164768	0.30378	0.48627
05 min	15	0.414361	0.097170	0.36055	0.46817
15 min	15	0.510791	0.227173	0.38499	0.63660
30 min	15	0.483262	0.139516	0.40600	0.56052

Means Comparisons

Comparisons for each pair using Student's t

Level	- Level	Difference	Lower CL	Upper CL	p-Value
15 min	Basal	0.1157664	-0.004240	0.2357731	0.0584
15 min	05 min	0.0964302	-0.023576	0.2164369	0.1131
30 min	Basal	0.0882380	-0.031769	0.2082446	0.1464
30 min	05 min	0.0689018	-0.051105	0.1889084	0.2550
15 min	30 min	0.0275284	-0.092478	0.1475351	0.6476
05 min	Basal	0.0193362	-0.100670	0.1393428	0.7481

A

Oneway Analysis of E2 By Time PCR=Promoter

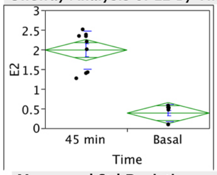

Means and Std Deviations

Level	Number	Mean	Std Dev	Std Err Mean
45 min	9	1.98689	0.487909	0.16264
Basal	9	0.38489	0.216937	0.07231

t Test

Basal-45 min

Assuming unequal variances

Difference	-1.6020	t Ratio	-9.00062
Std Err Dif	0.1780	DF	11.04412
Upper CL Dif	-1.2104	Prob > |t|	<.0001*
Lower CL Dif	-1.9936	Prob > t	1.0000
Confidence	0.95	Prob < t	<.0001*

Oneway Analysis of E2+RNAse H1 By Time PCR=Promoter

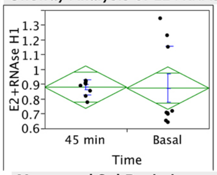

Matched Pairs 45 min

Wilcoxon Sign-Rank

	E2+RNAse H1-E2
Test Statistic	-22.500*
Prob > |z|	0.0039*
Prob > z	0.9980
Prob < z	0.0020*

Means and Std Deviations

Level	Number	Mean	Std Dev	Std Err Mean
45 min	9	0.879222	0.051088	0.01703
Basal	9	0.872778	0.282484	0.09416

t Test

Basal-45 min

Assuming unequal variances

Difference	-0.00644	t Ratio	-0.06735
Std Err Dif	0.09569	DF	8.522758
Upper CL Dif	0.21188	Prob > |t|	0.9479
Lower CL Dif	-0.22477	Prob > t	0.5261
Confidence	0.95	Prob < t	0.4739

Oneway Analysis of E2 By Time PCR=PolyA

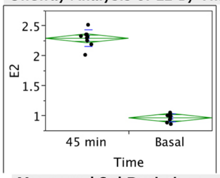

Means and Std Deviations

Level	Number	Mean	Std Dev	Std Err Mean
45 min	9	2.28700	0.136135	0.04538
Basal	9	0.96111	0.063623	0.02121

t Test

Basal-45 min

Assuming unequal variances

Difference	-1.3259	t Ratio	-26.4704
Std Err Dif	0.0501	DF	11.33554
Upper CL Dif	-1.2160	Prob > |t|	<.0001*
Lower CL Dif	-1.4357	Prob > t	1.0000
Confidence	0.95	Prob < t	<.0001*

Oneway Analysis of E2+RNAse H1 By Time PCR=PolyA

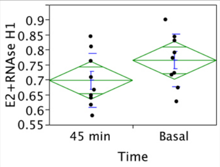

Matched Pairs 45 min

Wilcoxon Sign-Rank

	E2+RNAse H1-E2
Test Statistic	-22.500*
Prob > |z|	0.0039*
Prob > z	0.9980
Prob < z	0.0020*

Means and Std Deviations

Level	Number	Mean	Std Dev	Std Err Mean
45 min	9	0.698000	0.090623	0.03021
Basal	9	0.764667	0.087285	0.02910

t Test

Basal-45 min

Assuming unequal variances

Difference	0.06667	t Ratio	1.589543
Std Err Dif	0.04194	DF	15.97753
Upper CL Dif	0.15559	Prob > |t|	0.1315
Lower CL Dif	-0.02225	Prob > t	0.0658

Oneway Analysis of E2 By Time PCR=Control

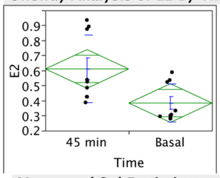

Means and Std Deviations

Level	Number	Mean	Std Dev	Std Err Mean
45 min	9	0.610667	0.224050	0.07468
Basal	9	0.383889	0.126650	0.04222

t Test

Basal-45 min

Assuming unequal variances

Difference	-0.22678	t Ratio	-2.64342
Std Err Dif	0.08579	DF	12.63892
Upper CL Dif	-0.04090	Prob > |t|	0.0207*
Lower CL Dif	-0.41265	Prob > t	0.9897
Confidence	0.95	Prob < t	0.0103*

Oneway Analysis of E2+RNAse H1 By Time PCR=Control

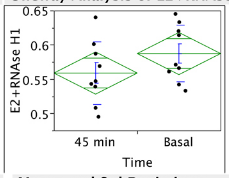

Matched Pairs 45 min

Wilcoxon Sign-Rank

	E2+RNAse H1-E2
Test Statistic	-1.5000*
Prob > |z|	0.9102
Prob > z	0.5449
Prob < z	0.4551

Means and Std Deviations

Level	Number	Mean	Std Dev	Std Err Mean
45 min	9	0.558778	0.045697	0.01523
Basal	9	0.587222	0.041249	0.01375

t Test

Basal-45 min

Assuming unequal variances

Difference	0.02844	t Ratio	1.386181
Std Err Dif	0.02052	DF	15.83508
Upper CL Dif	0.07198	Prob > |t|	0.1849
Lower CL Dif	-0.01509	Prob > t	0.0925
Confidence	0.95	Prob < t	0.9075

B

Oneway Analysis of oligo 1-9 By Treatment

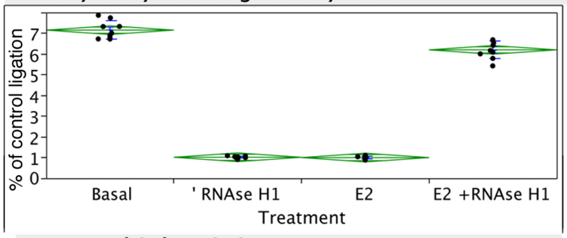

Means and Std Deviations

Level	Number	Mean	Std Dev	Std Err Mean
Basal	9	7.14400	0.43744	0.14581
' RNAse H1	9	1.01333	0.05454	0.01818
E2	9	0.99889	0.08223	0.02741
E2 +RNAse H1	9	6.19222	0.42493	0.14164

Means Comparisons

Comparisons for each pair using Student's t

Level	- Level	Difference	Lower CL	Upper CL	p-Value
Basal	E2	6.14511	5.84850	6.44172	<.0001*
Basal	' RNAse H1	6.13067	5.83406	6.42727	<.0001*
E2 +RNAse H1	E2	5.19333	4.89673	5.48994	<.0001*
E2 +RNAse H1	' RNAse H1	5.17889	4.88228	5.47550	<.0001*
Basal	E2 +RNAse H1	0.95178	0.65517	1.24838	<.0001*
' RNAse H1	E2	0.01444	-0.2822	0.31105	0.9216

Oneway Analysis of oligo 3-7 By Treatment

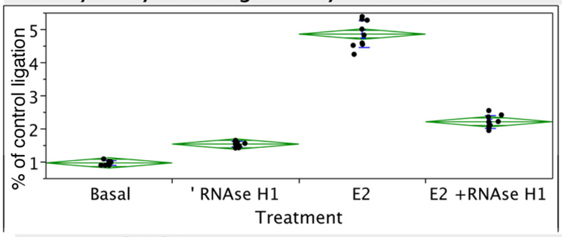

Means and Std Deviations

Level	Number	Mean	Std Dev	Std Err Mean
Basal	9	0.96056	0.06788	0.02263
' RNAse H1	9	1.52889	0.08038	0.02679
E2	9	4.84889	0.40925	0.13642
E2 +RNAse H1	9	2.20333	0.19742	0.06581

Means Comparisons

Comparisons for each pair using Student's t

Level	- Level	Difference	Lower CL	Upper CL	p-Value
E2	Basal	3.88833	3.66441	4.11226	<.0001*
E2	' RNAse H1	3.32000	3.09608	3.54392	<.0001*
E2	E2 +RNAse H1	2.64556	2.42163	2.86948	<.0001*
E2 +RNAse H1	Basal	1.24278	1.01885	1.46670	<.0001*
E2 +RNAse H1	' RNAse H1	0.67444	0.45052	0.89837	<.0001*
' RNAse H1	Basal	0.56833	0.34441	0.79226	<.0001*

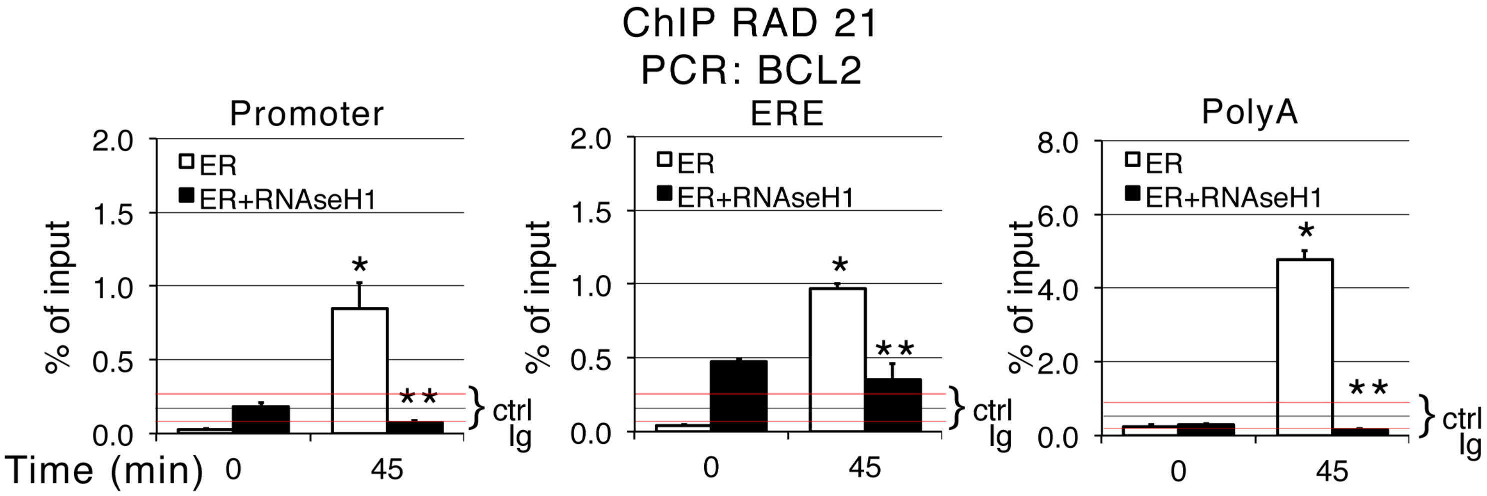
ChIP RAD 21
PCR: BCL2
Promoter
ERE
PolyA
ER
ER+RNAseH1
% of input
2.0
1.5
1.0
0.5
0.0
8.0
6.0
4.0
2.0
*
**
ctrl
Ig
Time (min)
0
45

A

Oneway Analysis of Oligo 3-7 +E2 By time

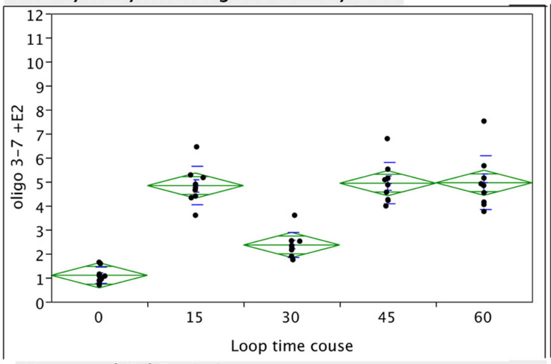

Oneway Analysis of oligo 3-7 +E2 +RNASE By time

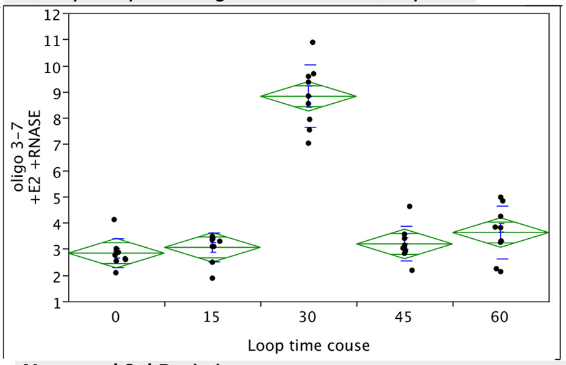

Means and Std Deviations (oligo 3-7 +E2)

Level	Number	Mean	Std Dev	Std Err Mean
0	9	1.10025	0.32396	0.10799
15	9	4.83556	0.78521	0.26174
30	9	2.36333	0.53167	0.17722
45	9	4.93556	0.85620	0.28540
60	9	4.95333	1.12898	0.37633

Means Comparisons

Comparisons for each pair using Student's t

Level	- Level	Difference	Lower CL	Upper CL	p-Value
60	0	3.853081	3.11363	4.592530	<.0001*
45	0	3.835303	3.09585	4.574753	<.0001*
15	0	3.735303	2.99585	4.474753	<.0001*
60	30	2.590000	1.85055	3.329450	<.0001*
45	30	2.572222	1.83277	3.311672	<.0001*
15	30	2.472222	1.73277	3.211672	<.0001*
30	0	1.263081	0.52363	2.002530	0.0013*
60	15	0.117778	-0.62167	0.857227	0.7492
45	15	0.100000	-0.63945	0.839450	0.7860
60	45	0.017778	-0.72167	0.757227	0.9615

Means and Std Deviations (oligo 3-7 +E2 +RNASE)

Level	Number	Mean	Std Dev	Std Err Mean
0	9	2.83422	0.55143	0.18381
15	9	3.05556	0.53437	0.17812
30	9	8.82000	1.20145	0.40048
45	9	3.18667	0.66280	0.22093
60	9	3.62222	1.00716	0.33572

Means Comparisons

Comparisons for each pair using Student's t

Level	- Level	Difference	Lower CL	Upper CL	p-Value
30	0	5.985784	5.19017	6.781399	<.0001*
30	15	5.764444	4.96883	6.560060	<.0001*
30	45	5.633333	4.83772	6.428949	<.0001*
30	60	5.197778	4.40216	5.993393	<.0001*
60	0	0.788006	-0.00761	1.583621	0.0521
60	15	0.566667	-0.22895	1.362282	0.1578
60	45	0.435556	-0.36006	1.231171	0.2752
45	0	0.352450	-0.44317	1.148066	0.3760
15	0	0.221339	-0.57428	1.016955	0.5771
45	15	0.131111	-0.66450	0.926727	0.7408

Matched Pairs

Time minutes=0

Wilcoxon Sign-Rank

+E2 +RNASE-+E2

Test Statistic	22.5000
Prob > |z|	0.0039*
Prob > z	0.0020*
Prob < z	0.9980

Time minutes=15

Wilcoxon Sign-Rank

+E2 +RNASE-+E2

Test Statistic	-22.500*
Prob > |z|	0.0039*
Prob > z	0.9980
Prob < z	0.0020*

Time minutes=30

Wilcoxon Sign-Rank

+E2 +RNASE-+E2

Test Statistic	22.5000
Prob > |z|	0.0039*
Prob > z	0.0020*
Prob < z	0.9980

Time minutes=45

Wilcoxon Sign-Rank

+E2 +RNASE-+E2

Test Statistic	-22.500*
Prob > |z|	0.0039*
Prob > z	0.9980
Prob < z	0.0020*

Time minutes=60

Wilcoxon Sign-Rank

+E2 +RNASE-+E2

Test Statistic	-12.500*
Prob > |z|	0.1641
Prob > z	0.9180
Prob < z	0.0820

B

Oneway Analysis of E2 By Time PCR=Promoter

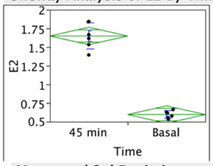

Means and Std Deviations

Level	Number	Mean	Std Dev	Std Err Mean
45 min	6	1.64917	0.175178	0.07152
Basal	6	0.59633	0.050007	0.02042

t Test

Basal-45 min

Assuming unequal variances

Difference	-1.0528	t Ratio	-14.1561
Std Err Dif	0.0744	DF	5.809509
Upper CL Dif	-0.8694	Prob > |t|	<.0001*
Lower CL Dif	-1.2363	Prob > t	1.0000
Confidence	0.95	Prob < t	<.0001*

Oneway Analysis of E2+RNAse H1 By Time PCR=Promoter

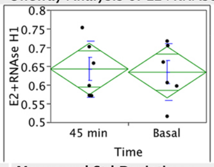

Matched Pairs 45 min

Wilcoxon Sign-Rank

E2+RNAse H1-E2

Test Statistic	-10.500*
Prob > |z|	0.0313*
Prob > z	0.9844
Prob < z	0.0156*

Means and Std Deviations

Level	Number	Mean	Std Dev	Std Err Mean
45 min	6	0.642333	0.074904	0.03058
Basal	6	0.633667	0.075809	0.03095

t Test

Basal-45 min

Assuming unequal variances

Difference	-0.00867	t Ratio	-0.1992
Std Err Dif	0.04351	DF	9.998558
Upper CL Dif	0.08828	Prob > |t|	0.8461
Lower CL Dif	-0.10561	Prob > t	0.5769
Confidence	0.95	Prob < t	0.4231

Oneway Analysis of E2 By Time PCR=ERE1

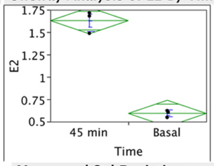

Means and Std Deviations

Level	Number	Mean	Std Dev	Std Err Mean
45 min	3	1.62833	0.123715	0.07143
Basal	3	0.59233	0.039323	0.02270

t Test

Basal-45 min

Assuming unequal variances

Difference	-1.0360	t Ratio	-13.8229
Std Err Dif	0.0749	DF	2.400046
Upper CL Dif	-0.7599	Prob > |t|	0.0024*
Lower CL Dif	-1.3121	Prob > t	0.9988
Confidence	0.95	Prob < t	0.0012*

Oneway Analysis of E2+RNAse H1 By Time PCR=ERE1

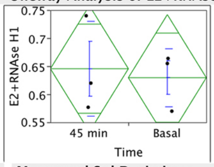

Matched Pairs 45 min

Wilcoxon Sign-Rank

E2+RNAse H1-E2

Test Statistic	-3.0000*
Prob > |z|	0.2500
Prob > z	0.8750
Prob < z	0.1250

Means and Std Deviations

Level	Number	Mean	Std Dev	Std Err Mean
45 min	3	0.645667	0.084477	0.04877
Basal	3	0.629667	0.051868	0.02995

t Test

Basal-45 min

Assuming unequal variances

Difference	-0.01600	t Ratio	-0.27956
Std Err Dif	0.05723	DF	3.320318
Upper CL Dif	0.15659	Prob > |t|	0.7963
Lower CL Dif	-0.18859	Prob > t	0.6018
Confidence	0.95	Prob < t	0.3982

Oneway Analysis of E2 By Time PCR=PolyA

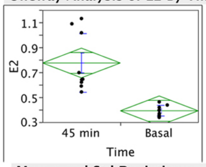

Means and Std Deviations

Level	Number	Mean	Std Dev	Std Err Mean
45 min	9	0.775000	0.233572	0.07786
Basal	9	0.391667	0.042697	0.01423

t Test

Basal-45 min

Assuming unequal variances

Difference	-0.38333	t Ratio	-4.84329
Std Err Dif	0.07915	DF	8.534049
Upper CL Dif	-0.20279	Prob > |t|	0.0011*
Lower CL Dif	-0.56388	Prob > t	0.9995
Confidence	0.95	Prob < t	0.0005*

Oneway Analysis of E2+RNAse H1 By Time PCR=PolyA

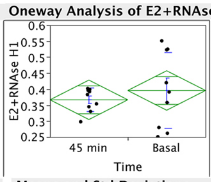

Matched Pairs 45 min

Wilcoxon Sign-Rank

E2+RNAse H1-E2

Test Statistic	-22.500*
Prob > |z|	0.0039*
Prob > z	0.9980
Prob < z	0.0020*

Means and Std Deviations

Level	Number	Mean	Std Dev	Std Err Mean
45 min	9	0.366556	0.036661	0.01222
Basal	9	0.395667	0.117939	0.03931

t Test

Basal-45 min

Assuming unequal variances

Difference	0.02911	t Ratio	0.707123
Std Err Dif	0.04117	DF	9.531724
Upper CL Dif	0.12145	Prob > |t|	0.4964
Lower CL Dif	-0.06323	Prob > t	0.2482
Confidence	0.95	Prob < t	0.7518

Oneway Analysis of E2 By Time PCR=Control

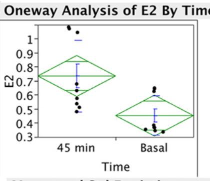

Means and Std Deviations

Level	Number	Mean	Std Dev	Std Err Mean
45 min	9	0.734778	0.256011	0.08534
Basal	9	0.452444	0.140985	0.04700

t Test

Basal-45 min

Assuming unequal variances

Difference	-0.28233	t Ratio	-2.89806
Std Err Dif	0.09742	DF	12.44362
Upper CL Dif	-0.07091	Prob > |t|	0.0129*
Lower CL Dif	-0.49376	Prob > t	0.9935
Confidence	0.95	Prob < t	0.0065*

Oneway Analysis of E2+RNAse H1 By Time PCR=Control

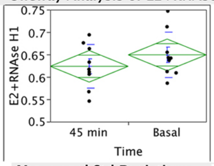

Matched Pairs 45 min

Wilcoxon Sign-Rank

E2+RNAse H1-E2

Test Statistic	-1.5000*
Prob > |z|	0.9102
Prob > z	0.5449
Prob < z	0.4551

Means and Std Deviations

Level	Number	Mean	Std Dev	Std Err Mean
45 min	9	0.623667	0.048836	0.01628
Basal	9	0.649222	0.050960	0.01699

t Test

Basal-45 min

Assuming unequal variances

Difference	0.02556	t Ratio	1.086193
Std Err Dif	0.02353	DF	15.97109
Upper CL Dif	0.07544	Prob > |t|	0.2935
Lower CL Dif	-0.02433	Prob > t	0.1468
Confidence	0.95	Prob < t	0.8532

Supplementary Figure 5

A

Oneway Analysis of ERE1 By TIME

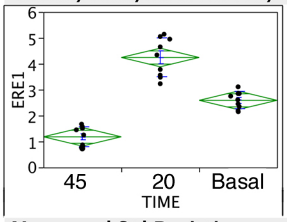

Means and Std Deviations

Level	Number	Mean	Std Dev	Std Err Mean
'+E 45 min	9	1.18222	0.385901	0.12863
+E 20 min	9	4.24222	0.743300	0.24777
Basal	9	2.58889	0.329865	0.10996

Means Comparisons

Comparisons for each pair using Student's t

Level	- Level	Difference	Lower CL	Upper CL	p-Value
+E 20 min	'+E 45 min	3.060000	2.554380	3.565620	<.0001*
+E 20 min	Basal	1.653333	1.147713	2.158953	<.0001*
Basal	'+E 45 min	1.406667	0.901047	1.912287	<.0001*

Oneway Analysis of ERE2 By TIME

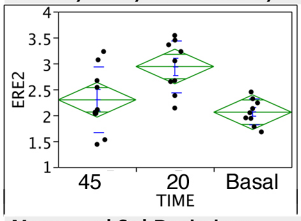

Means and Std Deviations

Level	Number	Mean	Std Dev	Std Err Mean
'+E 45 min	9	2.29900	0.627054	0.20902
+E 20 min	9	2.94111	0.500411	0.16680
Basal	9	2.06000	0.251595	0.08386

Means Comparisons

Comparisons for each pair using Student's t

Level	- Level	Difference	Lower CL	Upper CL	p-Value
+E 20 min	Basal	0.8811111	0.408828	1.353395	0.0008*
+E 20 min	'+E 45 min	0.6421111	0.169828	1.114395	0.0098*
'+E 45 min	Basal	0.2390000	-0.233284	0.711284	0.3067

Oneway Analysis of ERE3 By TIME

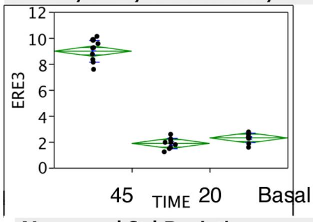

Means and Std Deviations

Level	Number	Mean	Std Dev	Std Err Mean
'+E 45 min	9	8.97889	0.842117	0.28071
+E 20 min	9	1.87889	0.415224	0.13841
Basal	9	2.30333	0.376630	0.12554

Means Comparisons

Comparisons for each pair using Student's t

Level	- Level	Difference	Lower CL	Upper CL	p-Value
'+E 45 min	+E 20 min	7.100000	6.53174	7.668262	<.0001*
'+E 45 min	Basal	6.675556	6.10729	7.243818	<.0001*
Basal	+E 20 min	0.424444	-0.14382	0.992707	0.1363

B

Oneway Analysis of E2 By Time PCR=ERE1

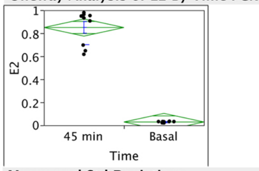

Means and Std Deviations

Level	Number	Mean	Std Dev	Std Err Mean
45 min	9	0.849444	0.149287	0.04976
Basal	9	0.029444	0.005940	0.00198

t Test

Basal-45 min

Assuming unequal variances

Difference	-0.82000	t Ratio	-16.4653
Std Err Dif	0.04980	DF	8.025327
Upper CL Dif	-0.70522	Prob > |t|	<.0001*
Lower CL Dif	-0.93478	Prob > t	1.0000
Confidence	0.95	Prob < t	<.0001*

Oneway Analysis of E2+RNAse H1 By Time PCR=ERE1

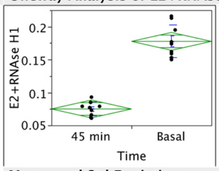

Matched Pairs 45 min

Wilcoxon Sign-Rank

	E2+RNAse H1-E2
Test Statistic	-22.500*
Prob > |z|	0.0039*
Prob > z	0.9980
Prob < z	0.0020*

Means and Std Deviations

Level	Number	Mean	Std Dev	Std Err Mean
45 min	9	0.075000	0.010665	0.00356
Basal	9	0.177667	0.025000	0.00833

t Test

Basal-45 min

Assuming unequal variances

Difference	0.102667	t Ratio	11.33188
Std Err Dif	0.009060	DF	10.81864
Upper CL Dif	0.122648	Prob > |t|	<.0001*
Lower CL Dif	0.082685	Prob > t	<.0001*
Confidence	0.95	Prob < t	1.0000

Oneway Analysis of E2 By Time PCR=PolyA

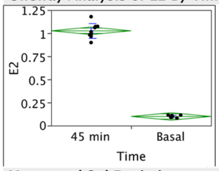

Means and Std Deviations

Level	Number	Mean	Std Dev	Std Err Mean
45 min	9	1.02622	0.081000	0.02700
Basal	9	0.09911	0.011921	0.00397

t Test

Basal-45 min

Assuming unequal variances

Difference	-0.92711	t Ratio	-33.9716
Std Err Dif	0.02729	DF	8.3464
Upper CL Dif	-0.86463	Prob > |t|	<.0001*
Lower CL Dif	-0.98959	Prob > t	1.0000
Confidence	0.95	Prob < t	<.0001*

Oneway Analysis of E2+RNAse H1 By Time PCR=PolyA

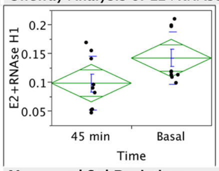

Matched Pairs 45 min

Wilcoxon Sign-Rank

	E2+RNAse H1-E2
Test Statistic	-22.500*
Prob > |z|	0.0039*
Prob > z	0.9980
Prob < z	0.0020*

Means and Std Deviations

Level	Number	Mean	Std Dev	Std Err Mean
45 min	9	0.098333	0.046373	0.01546
Basal	9	0.141889	0.045584	0.01519

t Test

Basal-45 min

Assuming unequal variances

Difference	0.04356	t Ratio	2.009457
Std Err Dif	0.02168	DF	15.99528
Upper CL Dif	0.08951	Prob > |t|	0.0617
Lower CL Dif	-0.00240	Prob > t	0.0308*
Confidence	0.95	Prob < t	0.9692

Oneway Analysis of E2 By Time PCR=Intron

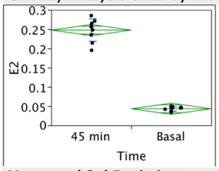

Means and Std Deviations

Level	Number	Mean	Std Dev	Std Err Mean
45 min	9	0.247444	0.028465	0.00949
Basal	9	0.043111	0.005925	0.00198

t Test

Basal-45 min

Assuming unequal variances

Difference	-0.20433	t Ratio	-21.083
Std Err Dif	0.00969	DF	8.692016
Upper CL Dif	-0.18229	Prob > |t|	<.0001*
Lower CL Dif	-0.22638	Prob > t	1.0000
Confidence	0.95	Prob < t	<.0001*

Oneway Analysis of E2+RNAse H1 By Time PCR=Intron

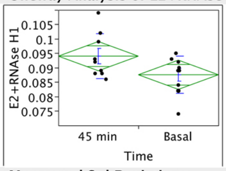

Means and Std Deviations

Level	Number	Mean	Std Dev	Std Err Mean
45 min	9	0.094000	0.007746	0.00258
Basal	9	0.087556	0.006540	0.00218

t Test

Basal-45 min

Assuming unequal variances

Difference	-0.00644	t Ratio	-1.90703
Std Err Dif	0.00338	DF	15.56301
Upper CL Dif	0.00074	Prob > |t|	0.0751
Lower CL Dif	-0.01362	Prob > t	0.9624
Confidence	0.95	Prob < t	0.0376*

Oneway Analysis of E2 By Time PCR=Control

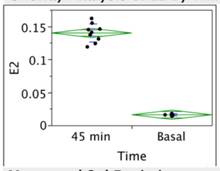

Means and Std Deviations

Level	Number	Mean	Std Dev	Std Err Mean
45 min	9	0.140000	0.013955	0.00465
Basal	9	0.016111	0.001167	0.00039

t Test

Basal-45 min

Assuming unequal variances

Difference	-0.12389	t Ratio	-26.5401
Std Err Dif	0.00467	DF	8.111819
Upper CL Dif	-0.11315	Prob > |t|	<.0001*
Lower CL Dif	-0.13463	Prob > t	1.0000
Confidence	0.95	Prob < t	<.0001*

Oneway Analysis of E2+RNAse H1 By Time PCR=Control

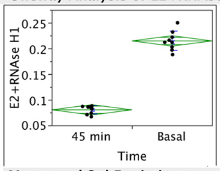

Means and Std Deviations

Level	Number	Mean	Std Dev	Std Err Mean
45 min	9	0.080444	0.007764	0.00259
Basal	9	0.214667	0.018635	0.00621

t Test

Basal-45 min

Assuming unequal variances

Difference	0.134222	t Ratio	19.94652
Std Err Dif	0.006729	DF	10.69614
Upper CL Dif	0.149084	Prob > |t|	<.0001*
Lower CL Dif	0.119360	Prob > t	<.0001*
Confidence	0.95	Prob < t	1.0000

Oneway Analysis of RNA Level By Treatment OLIGO=Oligo 10

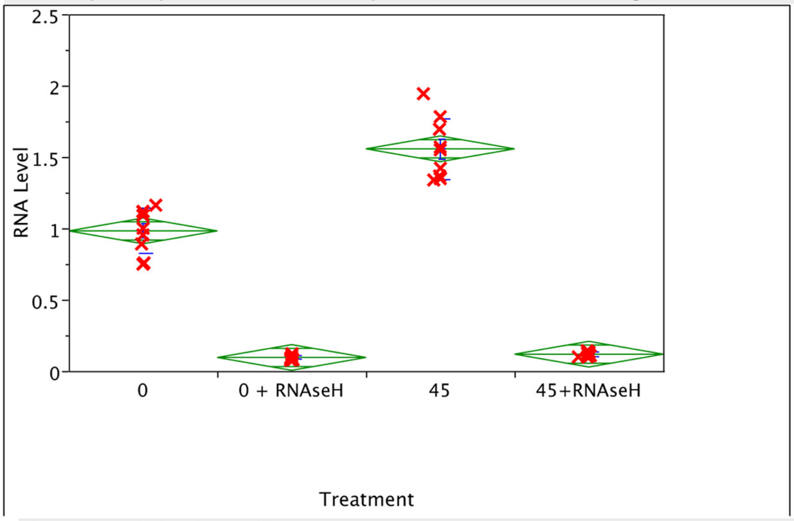

Means and Std Deviations

Level	Number	Mean	Std Dev	Std Err Mean
0	9	0.98189	0.155948	0.05198
0 + RNAseH	9	0.09511	0.014995	0.00500
45	9	1.55700	0.213420	0.07114
45+RNAseH	9	0.11822	0.015230	0.00508

Means Comparisons

Comparisons for each pair using Student's t

Level	- Level	Difference	Lower CL	Upper CL	p-Value
45	0 + RNAseH	1.461889	1.33457	1.589208	<.0001*
45	45+RNAseH	1.438778	1.31146	1.566097	<.0001*
0	0 + RNAseH	0.886778	0.75946	1.014097	<.0001*
0	45+RNAseH	0.863667	0.73635	0.990986	<.0001*
45	0	0.575111	0.44779	0.702430	<.0001*
45+RNAseH	0 + RNAseH	0.023111	-0.10421	0.150430	0.7140

Oneway Analysis of RNA Level By Treatment OLIGO=Oligo 7

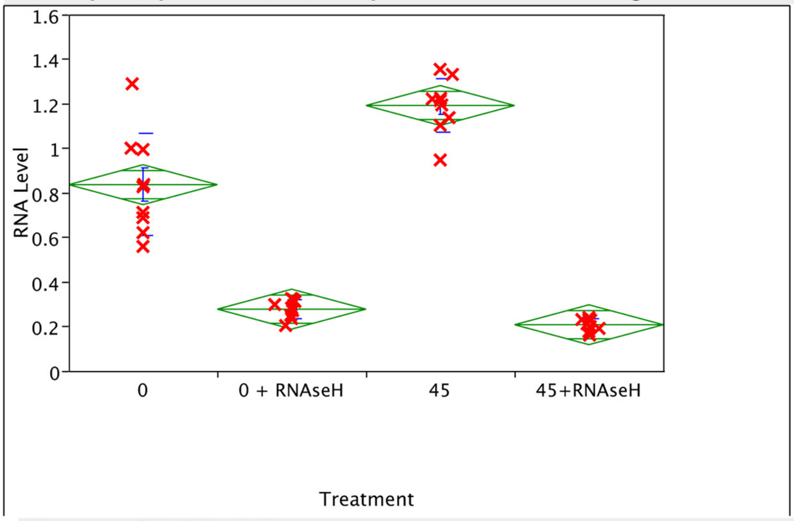

Means and Std Deviations

Level	Number	Mean	Std Dev	Std Err Mean
0	9	0.83633	0.228414	0.07614
0 + RNAseH	9	0.27767	0.041527	0.01384
45	9	1.19167	0.121906	0.04064
45+RNAseH	9	0.20767	0.027313	0.00910

Means Comparisons

Comparisons for each pair using Student's t

Level	- Level	Difference	Lower CL	Upper CL	p-Value
45	45+RNAseH	0.9840000	0.857425	1.110575	<.0001*
45	0 + RNAseH	0.9140000	0.787425	1.040575	<.0001*
0	45+RNAseH	0.6286667	0.502092	0.755241	<.0001*
0	0 + RNAseH	0.5586667	0.432092	0.685241	<.0001*
45	0	0.3553333	0.228759	0.481908	<.0001*
0 + RNAseH	45+RNAseH	0.0700000	-0.056575	0.196575	0.2683

Original Gels Displayed in Figure 1

Promoter-Enhancer 5’

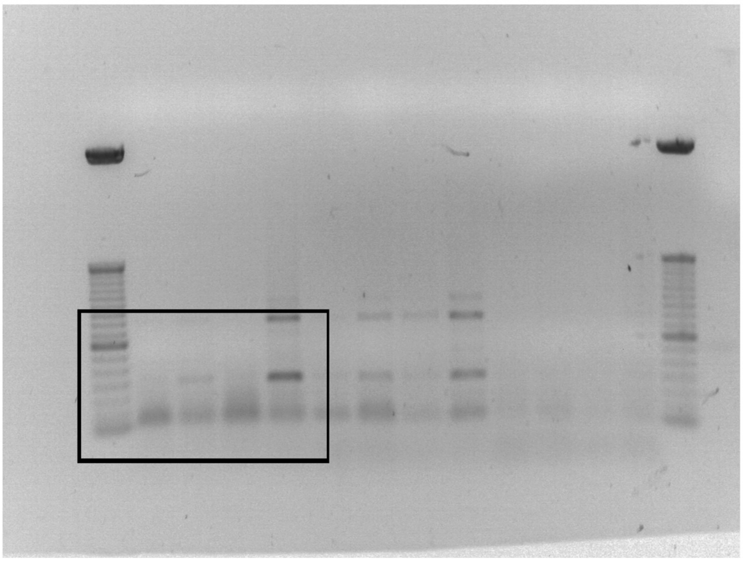

Promoter-PolyA

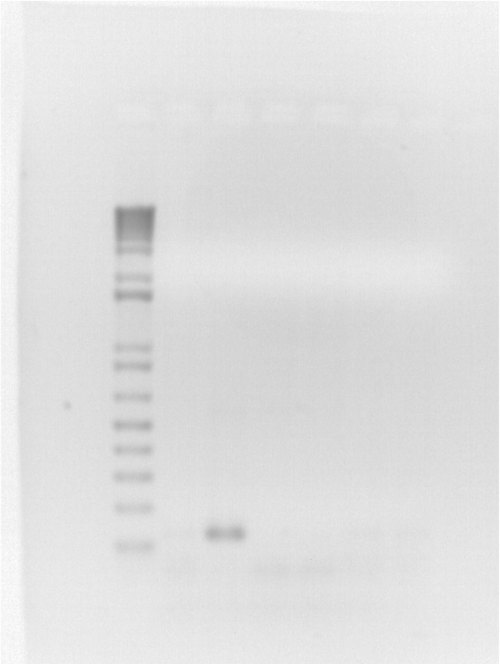

Enhancer 5’-PolyA

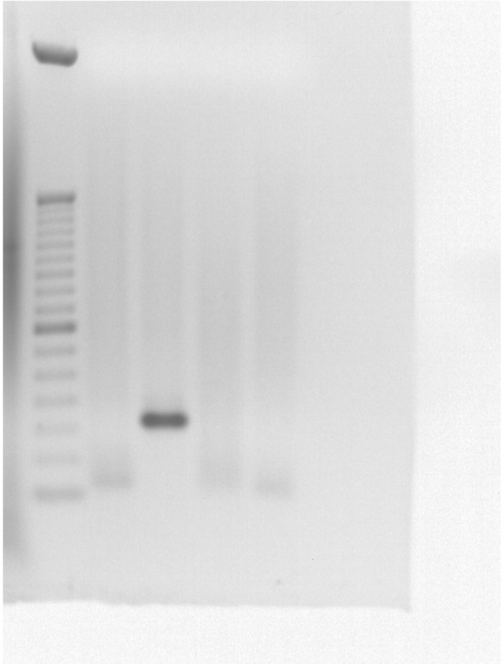

Promoter-Enhancer 3’

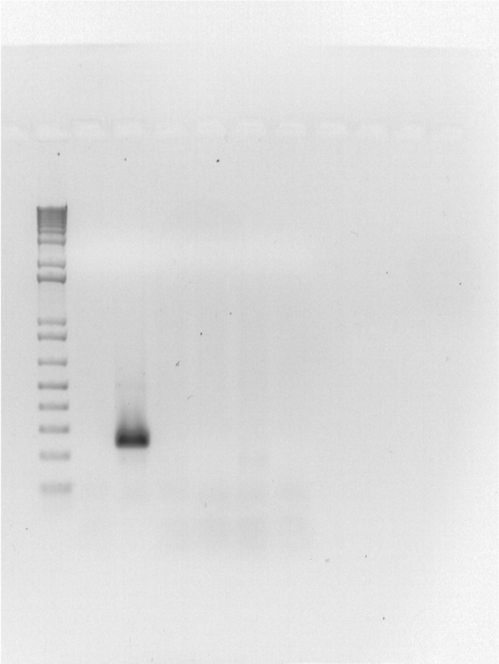

Original Gels Displayed in Figure 2

2B Upper Panel

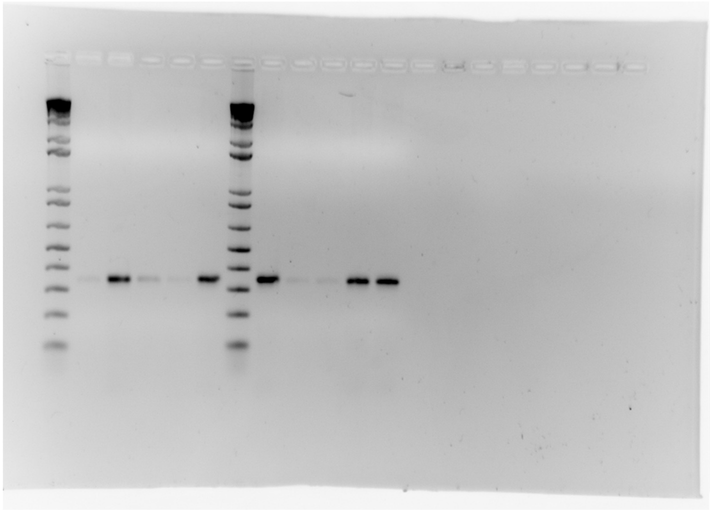

2B Lower Panel

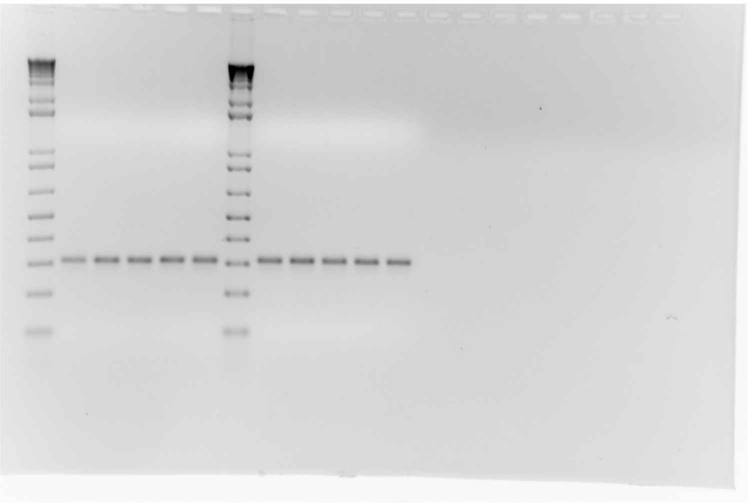

2D Upper Panel

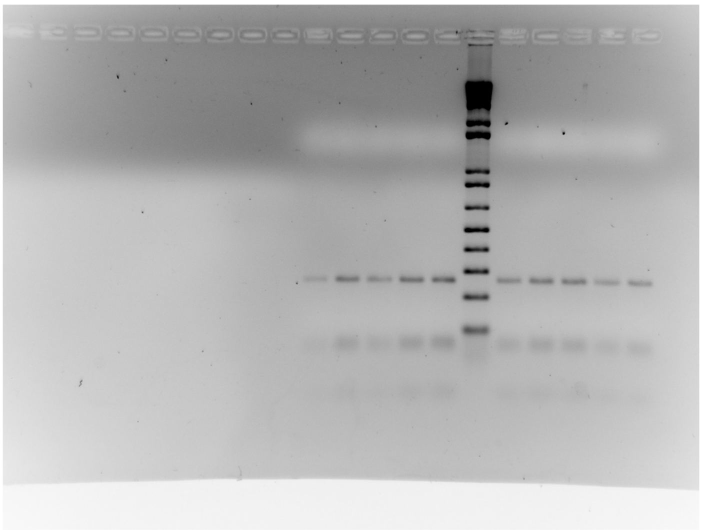

2D Lower Panel

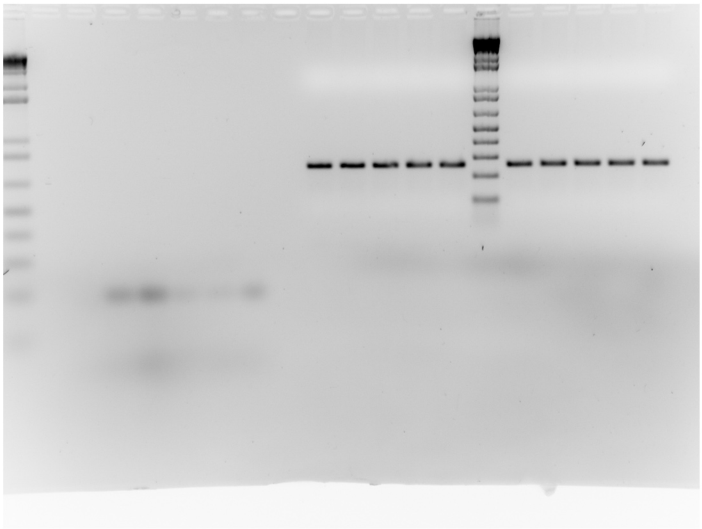

Supplement: Supplementary file 1 — Supplementary Data [file 41598_2019_40123_MOESM1_ESM.pdf]
